# Supplementary material for: Polysulfur-based bulking of dynamin-related protein 1 prevents ischemic sulfide catabolism and heart failure in mice
Source: Nat Commun. 2025 Jan 2;16:276. doi: 10.1038/s41467-024-55661-5 (PMC11695708; doi:10.1038/s41467-024-55661-5)
Supplement: Supplementary file 1 — Supplementary Information [file 41467_2024_55661_MOESM1_ESM.docx]

**Supplementary Information**

**Polysulfur-based bulking of dynamin-related protein 1 prevents ischemic sulfide catabolism and heart failure in mice**

**Authors:** Akiyuki Nishimura, Seiryo Ogata, Xiaokang Tang, Kowit Hengphasatporn, Keitaro Umezawa, Makoto Sanbo, Masumi Hirabayashi, Yuri Kato, Yuko Ibuki, Yoshito Kumagai, Kenta Kobayashi, Yasunari Kanda, Yasuteru Urano, Yasuteru Shigeta, Takaaki Akaike, Motohiro Nishida


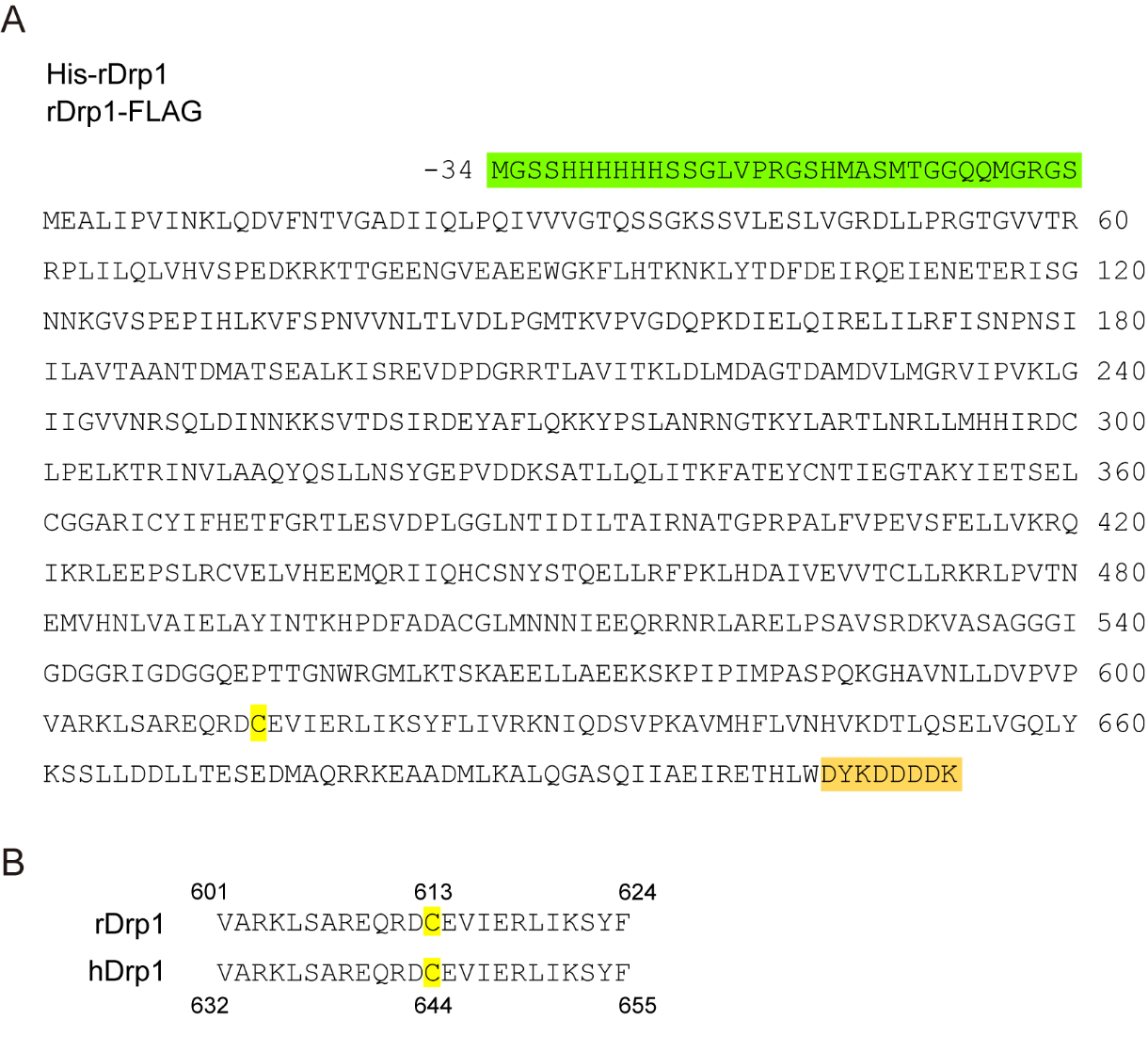


**Supplementary Fig. 1.** **Amino acid sequence of recombinant rat His-Drp1 and Drp1-FLAG for Mass spectrometry analysis.** (**A**) Rat Drp1 isoform X13 (XP_038943828.1) was fused with the N-terminal His-tag sequence (green highlight) or the C-terminal FLAG sequence (orange highlight). Redox-sensitive Cys613 is shown as a yellow highlight. (**B**) Amino acid alignment of His-rDrp1 and human Drp1 isoform 1 (NP_036192.2). Cys613 in which we identified polysulfidation by MASS spectrometry is consistent with Cys644 of human Drp1.


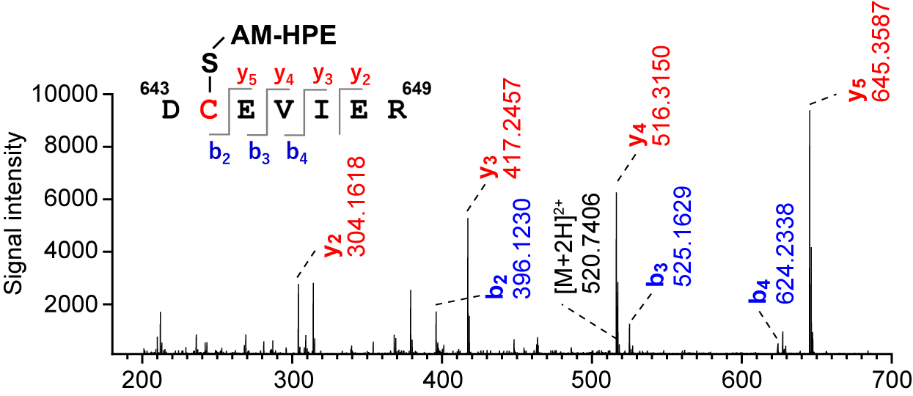


**Supplementary Fig. 2.** **MS/MS spectra for identifying CysSH of Cys644 in Drp1.** The identified fragment ions shown in the spectra and the peptide sequence (b and y ions) evidenced the presence of CysSH labeled with HPE-IAM (marked in red).

**
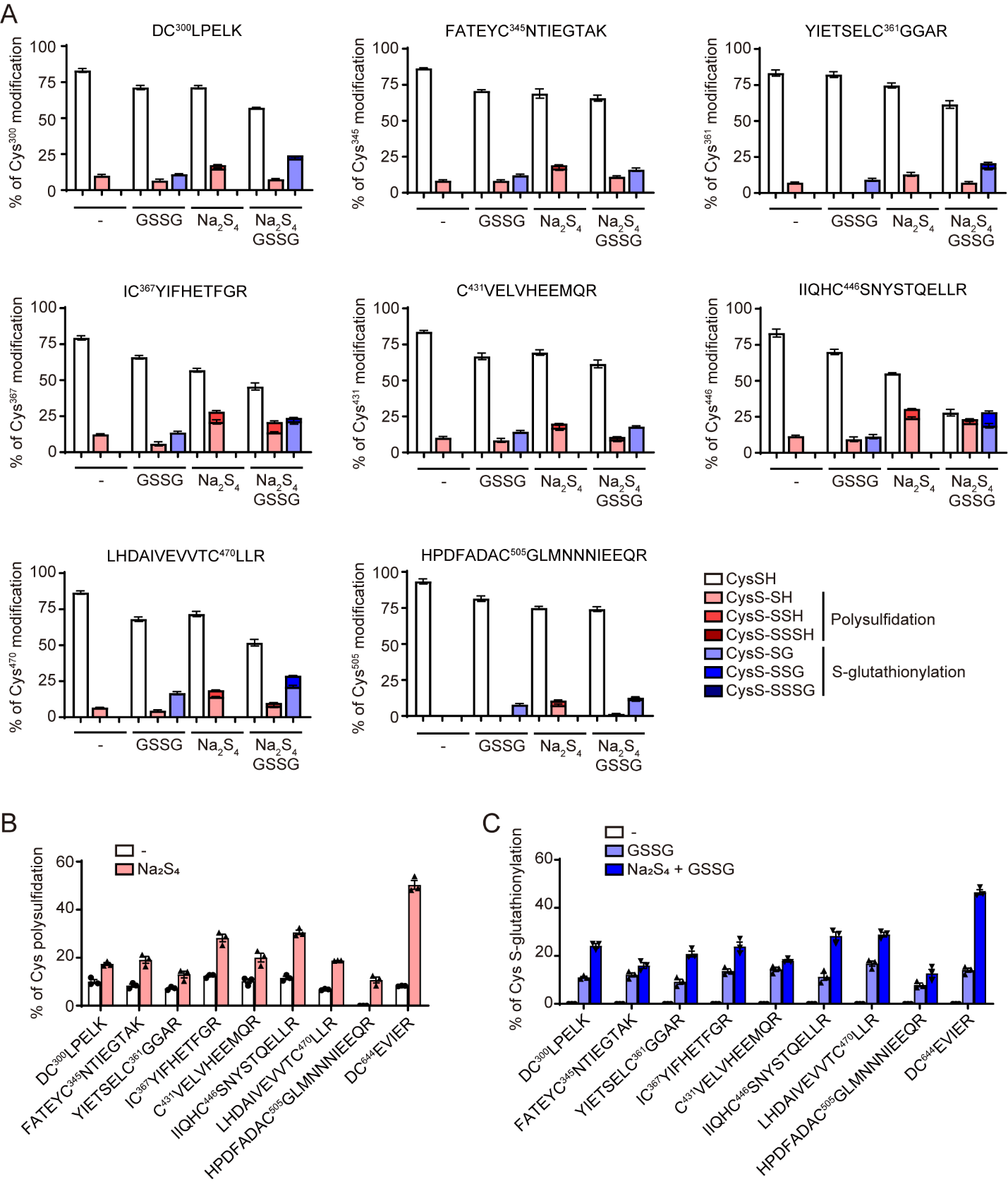
**

**Supplementary Fig. 3. Quantification of polysulfidation and S-glutathionylation of each cysteine in Drp1.** (**A**) His-Drp1 purified from *E. coli.* was reacted with or without Na_2_S_4_ to prepare highly polysulfidated Drp1, and then treated with or without GSSG (1 mM). The proportion of CysSH, polysulfidation (CysS-SH, CysS-SSH and CysS-SSSH) and S-glutathionylation (CysS-SG, CysS-SSG and CysS-SSSG) at each cysteine was quantified. (**B**, **C**) Comparison of polysulfidation (**B**) and S-glutathionylation (**C**) efficiency of each cysteine in Drp1 (n=3 independent experiments). Source data are provided as a Source Data file.

**
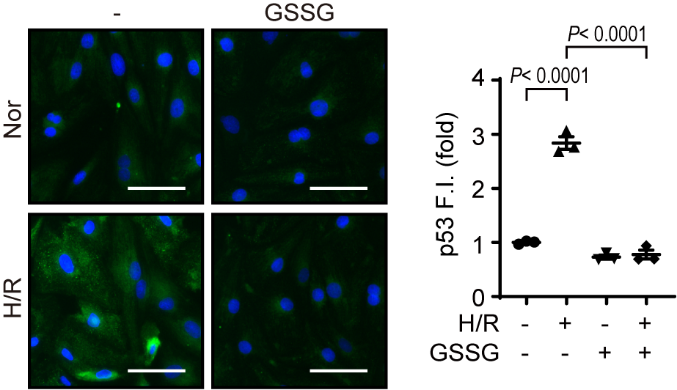
**

**Supplementary Fig. 4. Effect of GSSG treatment on p53-positive cellular senescence.** Representative images of p53 staining in NRCMs with or without GSSG under hypoxia/reoxygenation (H/R). Fluorescence intensity of p53 was quantified. (n=3 independent experiments). Scale bar, 50 µm. Data are shown as the means ± SEM. Significance was determined by one-way ANOVA followed by Tukey’s post-hoc test. Source data are provided as a Source Data file.


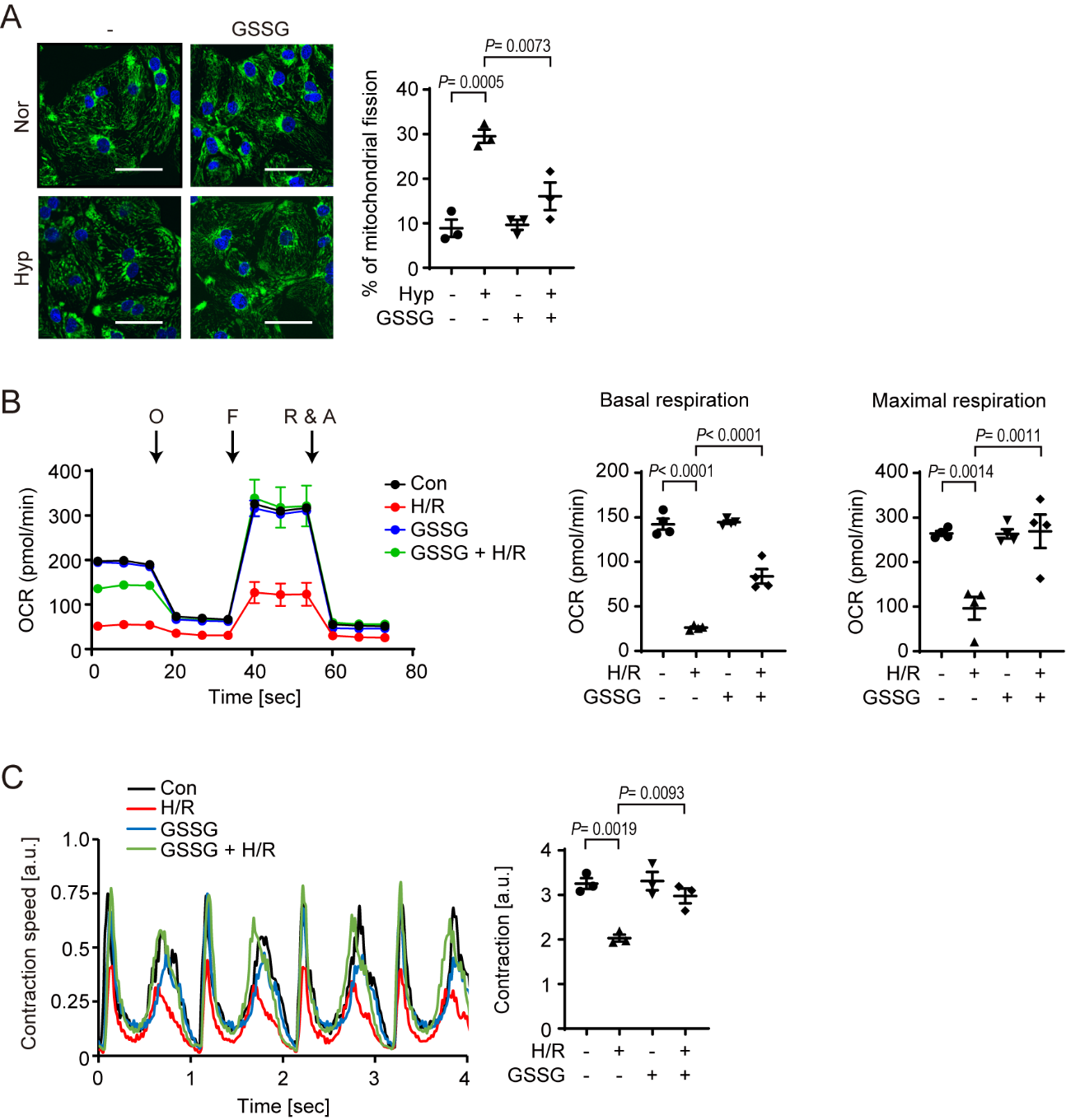


**Supplementary Fig. 5. Cardioprotective effect of GSSG for human cardiomyocytes.** (**A**) Representative images of mitochondrial morphology in hiPS-CMs pretreated with GSSG under normoxia (Nor) or hypoxia (Hyp). The percentage of cells with vesicle-type mitochondria was quantified. (n=3 independent experiments). Scale bars, 50 µm. (**B**) Oxygen consumption rate (OCR) in hiPS-CMs pretreated with GSSG under normoxia or hypoxia/reoxygenation (H/R). Oligomycin (O), FCCP (F), and rotenone + antimycin A (R & A) were added at the indicated timing. Right graphs show the quantitative analysis of basal and maximal respiration. (n=4 independent experiments). (**C**) Representative traces of contraction speed of hiPS-CMs under Nor or H/R. The contraction was calculated (n=3 independent experiments). Data are shown as the means ± SEM. Significance was determined by one-way ANOVA followed by Tukey’s post-hoc test. Source data are provided as a Source Data file.


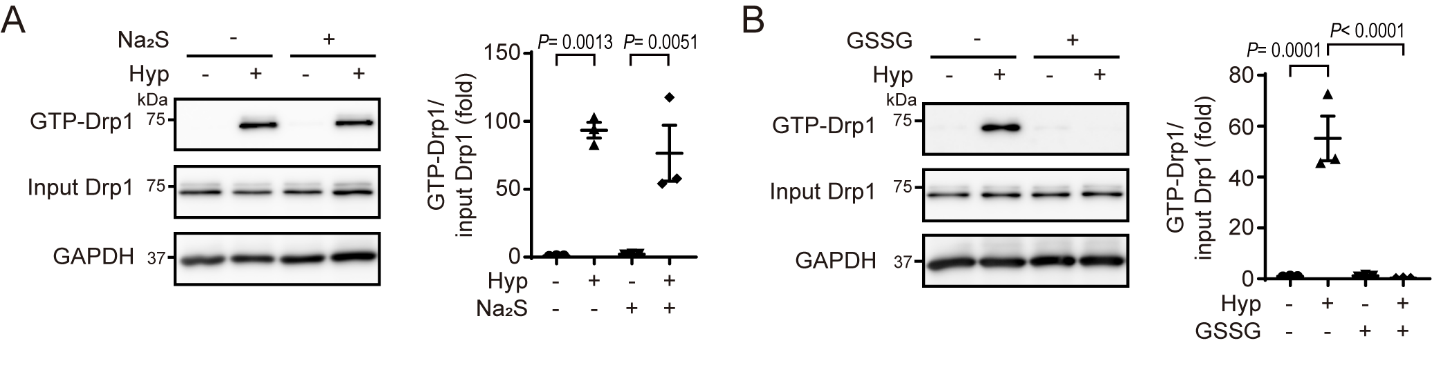


**Supplementary Fig. 6. The GTP-binding activity of Drp1 under hypoxia.** (**A**) Effect of Na_2_S pretreatment on hypoxia (Hyp)-induced Drp1 activation. (n=3 independent experiments). (**B**) Effect of GSSG short time pretreatment on Hyp-induced Drp1 activation. After 30 min pretreatment with GSSG, NRCMs were cultured under hypoxia for 1 h. (n=3 independent experiments). Data are shown as the means ± SEM. Significance was determined by one-way ANOVA followed by Tukey’s post-hoc test. Source data are provided as a Source Data file.

**
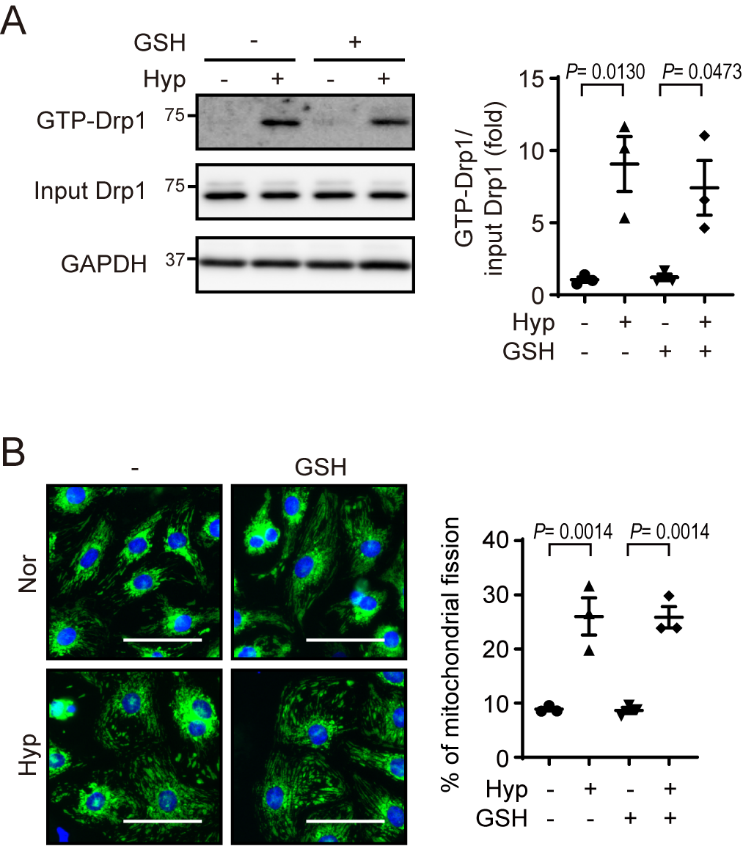
**

**Supplementary Fig. 7. Effect of GSH treatment on Drp1-mediated mitochondrial fission.** (**A**) Effect of GSH treatment on hypoxia (Hyp)-induced Drp1 activation. (n=3 independent experiments). (**B**) Representative images of mitochondrial morphology in NRCMs pretreated with GSH under normoxia (Nor) or Hyp. The percentage of cells with vesicle-type mitochondria was quantified. (n=3 independent experiments). Scale bars, 50 µm. Data are shown as the means ± SEM. Significance was determined by one-way ANOVA followed by Tukey’s post-hoc test. Source data are provided as a Source Data file.


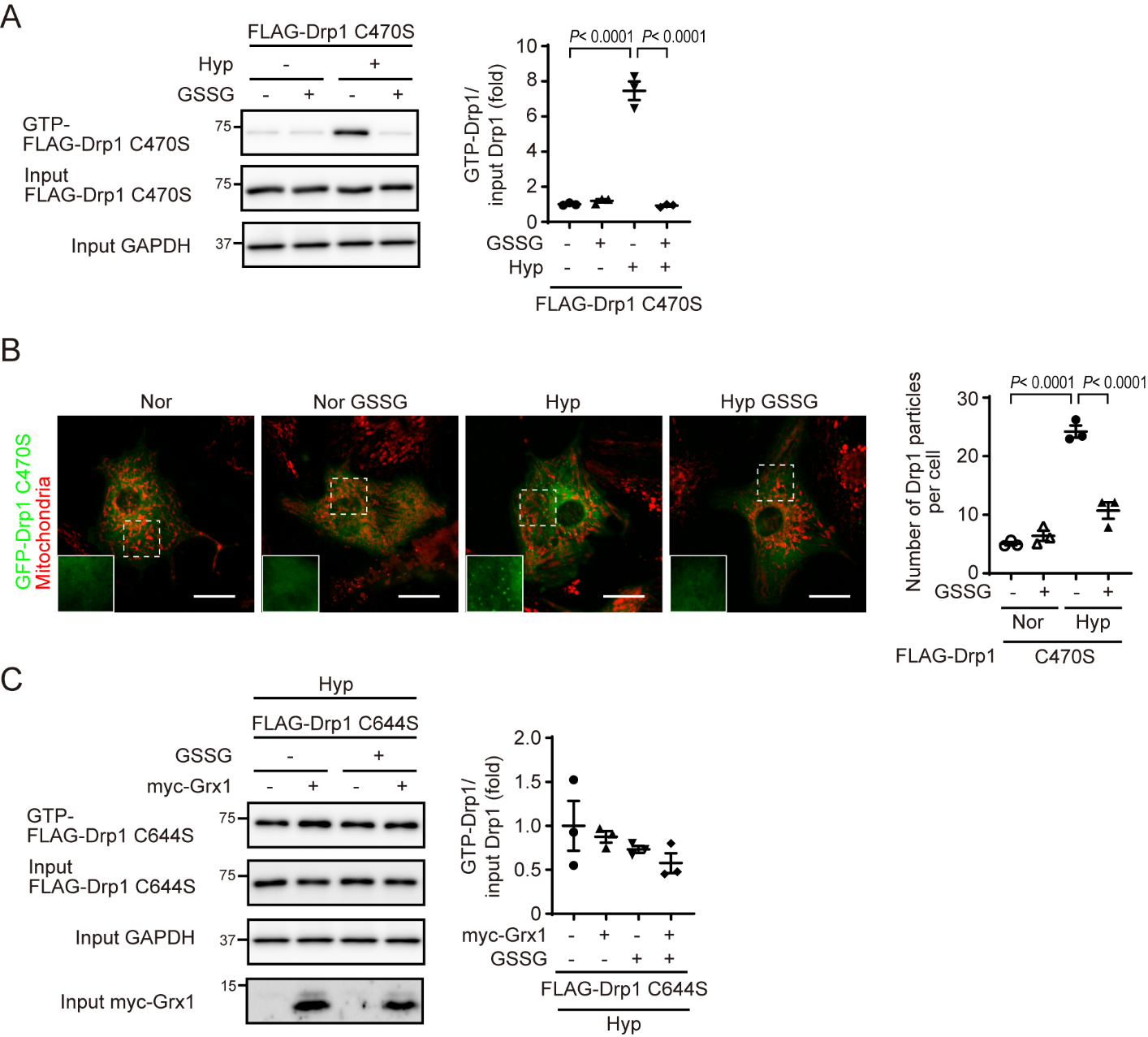


**Supplementary Fig. 8. Cys470 is not required for GSSG-induced Drp1 inhibition.** (**A**) Effect of GSSG treatment on the GTP-binding activity of Drp1 C470S. (n=3 independent experiments). (**B**) Representative images of GFP-Drp1 C470S (green) and mitochondria (red) in NRCMs treated with or without GSSG under normoxia (Nor) or hypoxia (Hyp). GFP-Drp1 C470S was transfected into NRCMs. The dashed square is enlarged and is shown as green single channel. The number of GFP-Drp1 particles per cell was quantified. (n=3 independent experiments). Scale bars, 20 µm. (**C**) Effect of Grx1 expression on GTP-binding activity of Drp1 C644S by GSSG under Hyp. (n=3 independent experiments). Data are shown as the means ± SEM. Significance was determined by one-way ANOVA followed by Tukey’s post-hoc test. Source data are provided as a Source Data file.


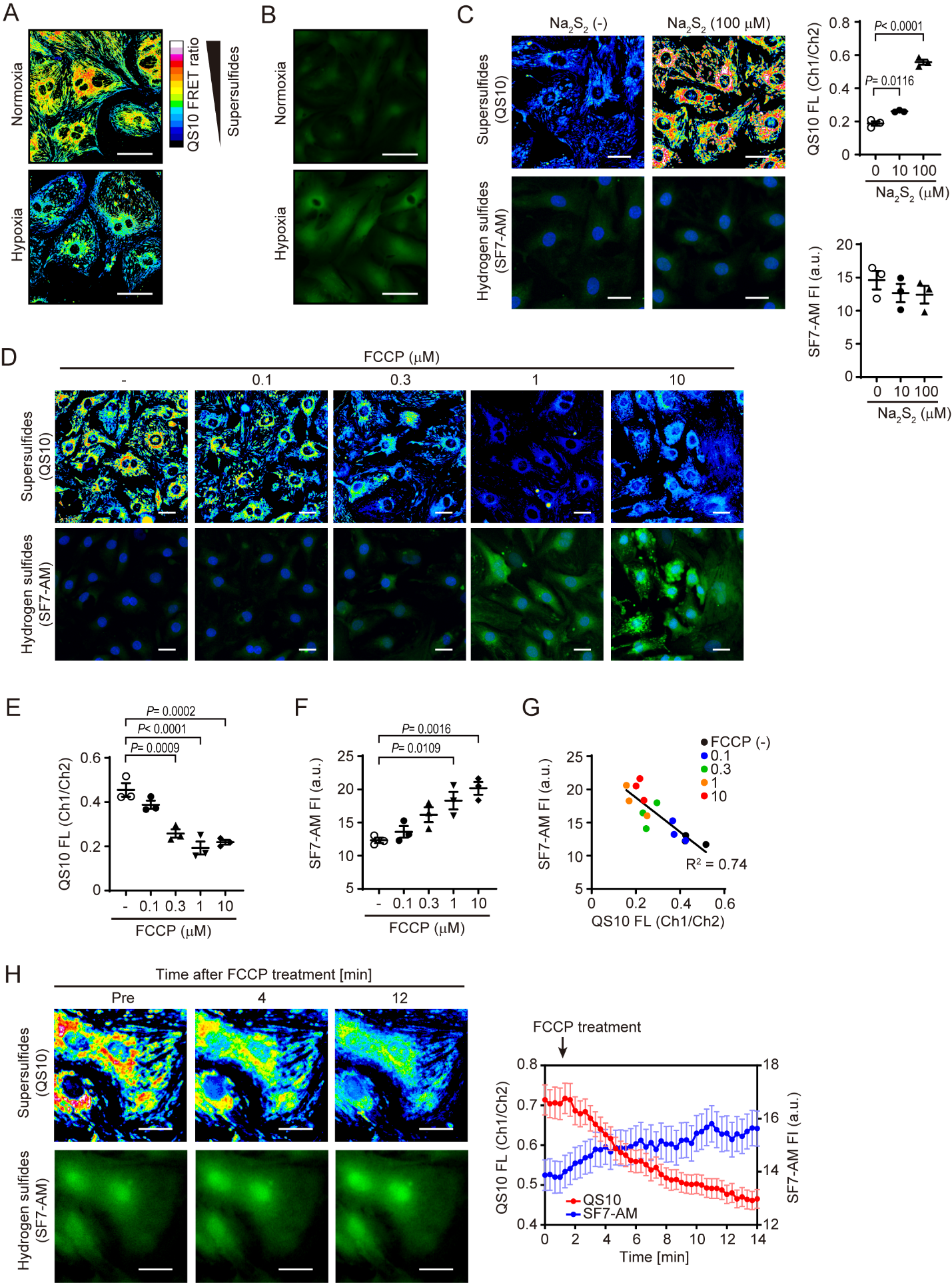


**Supplementary Fig. 9. Facilitation of supersulfide catabolism by hypoxia-mediated mitochondrial dysfunction.** (**A**, **B**) Representative images of supersulfides (**A**) and H_2_S (**B**). NRCMs were incubated in hypoxia for 18 h, and then loaded with QS10 or SF7-AM for supersulfide or hydrogen sulfide imaging, respectively. (n=3 independent experiments). Scale bar, 50 µm. (**C**) Simultaneous imaging of supersulfides and H_2_S and dose-dependent effect of supersulfide donor Na_2_S_2_. NRCMs that labeled with both QS10 and SF7-AM were incubated with the indicated concentration of Na_2_S_2_ for 5 min. Changes in QS10 fluorescence ratio and SF7-AM fluorescence intensity were quantified. Scale bar, 20 µm. (n=3 independent experiments). (**D**) Simultaneous imaging of supersulfides and H_2_S and dose-dependent effect of mitochondria uncoupler FCCP. NRCMs were incubated with the indicated concentration of FCCP for 20 min. Scale bar, 20 µm. (**E**-**G**) Changes in QS10 fluorescence ratio (**E**) and SF7-AM fluorescence intensity (**F**) by FCCP. Co-relation between QS10 fluorescence ratio and SF7-AM fluorescence intensity in FCCP-treated NRCMs (**G**). (n=3 independent experiments). (**H**) Simultaneous images of supersulfides and H_2_S in NRCMs before (Pre) and 4 and 12 min after 1 µM FCCP administration. Changes in QS10 fluorescence ratio (red) and SF7-AM fluorescence intensity (blue) during 1 µM FCCP treatment are shown in the right panel. (n=8 cells). Scale bar, 20 µm. Data are shown as the means ± SEM. Significance was determined by one-way ANOVA followed by Tukey’s post-hoc test. Source data are provided as a Source Data file.

**
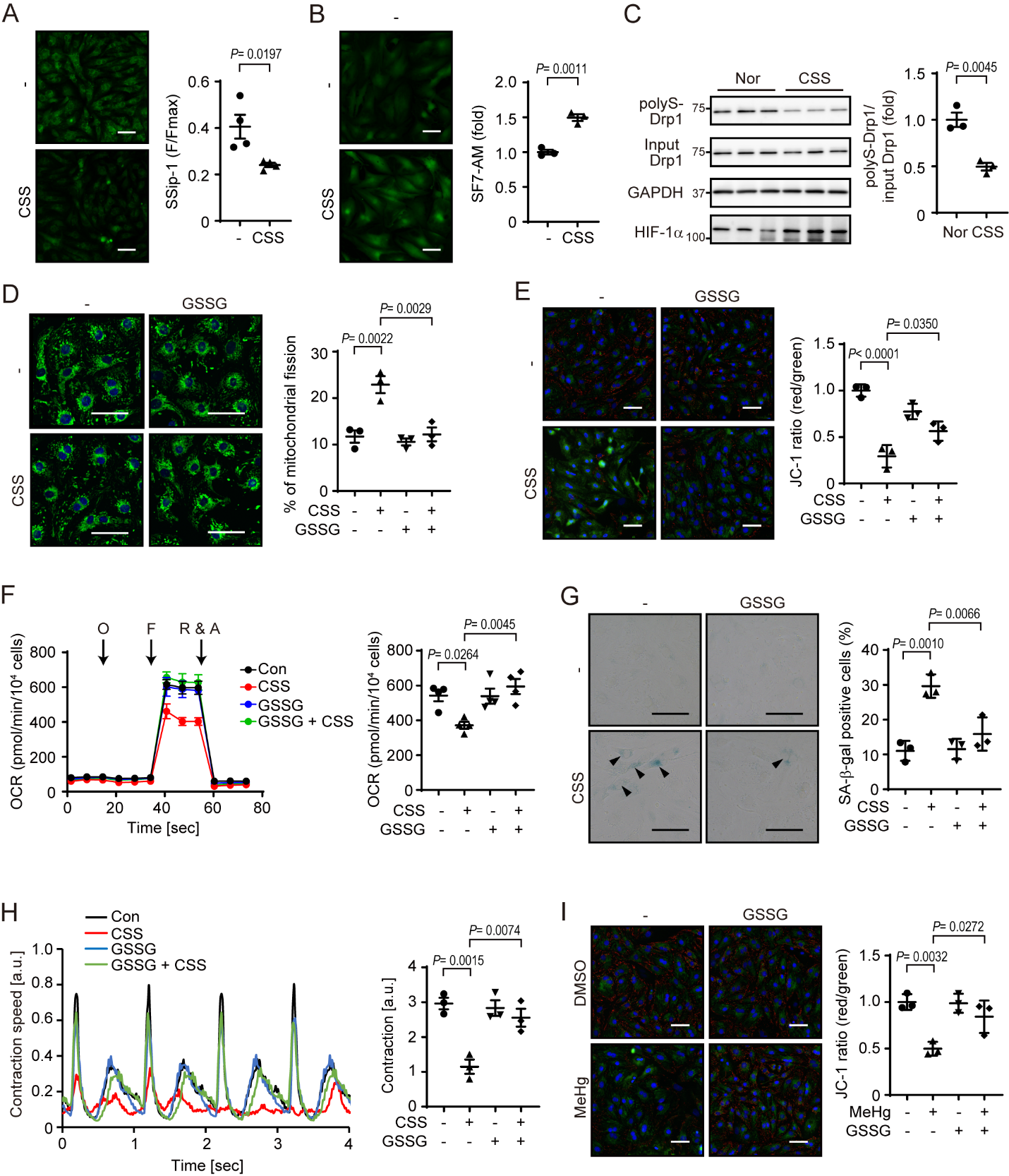
**

**Supplementary Fig. 10. Cigarette sidestream smoke (CSS)-mediated supersulfide catabolism induces Drp1 depolysulfidation-related myocardial dysfunction, which is inhibited by GSSG.** (**A**, **B**) Representative images of supersulfides (**A**) and H_2_S (**B**). NRCMs were incubated with CSS, and then loaded with SSip-1 (**A**) or SF7-AM (**B**). Fluorescence intensity for each cell was quantified (n=4 (**A**) or 3 (**B**) independent experiments). Scale bar, 50 µm. (**C**) Effect of CSS treatment on polysulfidation of Drp1. NRCMs were incubated with CSS, and Drp1 polysulfidation was analyzed (n=3 independent experiments). (**D**, **E**) Representative images of mitochondrial morphology (**D**) and mitochondrial membrane potential (**E**) in NRCMs treated with CSS and GSSG. The percentage of cells with vesicle-type mitochondria (**D**) and average JC-1 red/green ratio (**E**) was quantified. (n=3 independent experiments). Scale bars, 50 µm. (**F**) Oxygen consumption rate (OCR) in NRCMs. Oligomycin (O), FCCP (F), and rotenone + antimycin A (R & A) were added at the indicated timing. Right graphs show the quantitative analysis of maximal respiration. (n=4 independent experiments). (**G**) Representative images of SA-β-gal staining in NRCMs. The percentage of SA-β-gal positive cells was quantified. (n=3 independent experiments). Scale bars, 50 µm. (**H**) Representative traces of contraction speed of NRCMs. The contraction was calculated. (n=3 independent experiments). (**I**) Representative images of mitochondrial membrane potential in NRCMs treated with MeHg and GSSG. The average JC-1 red/green ratio was quantified. (n=3 independent experiments). Scale bars, 50 µm. Data are shown as the means ± SEM. Significance was determined by two-sided unpaired t-test (**A**-**C**); one-way ANOVA followed by Tukey’s post-hoc test (**D**-**I**). Source data are provided as a Source Data file.


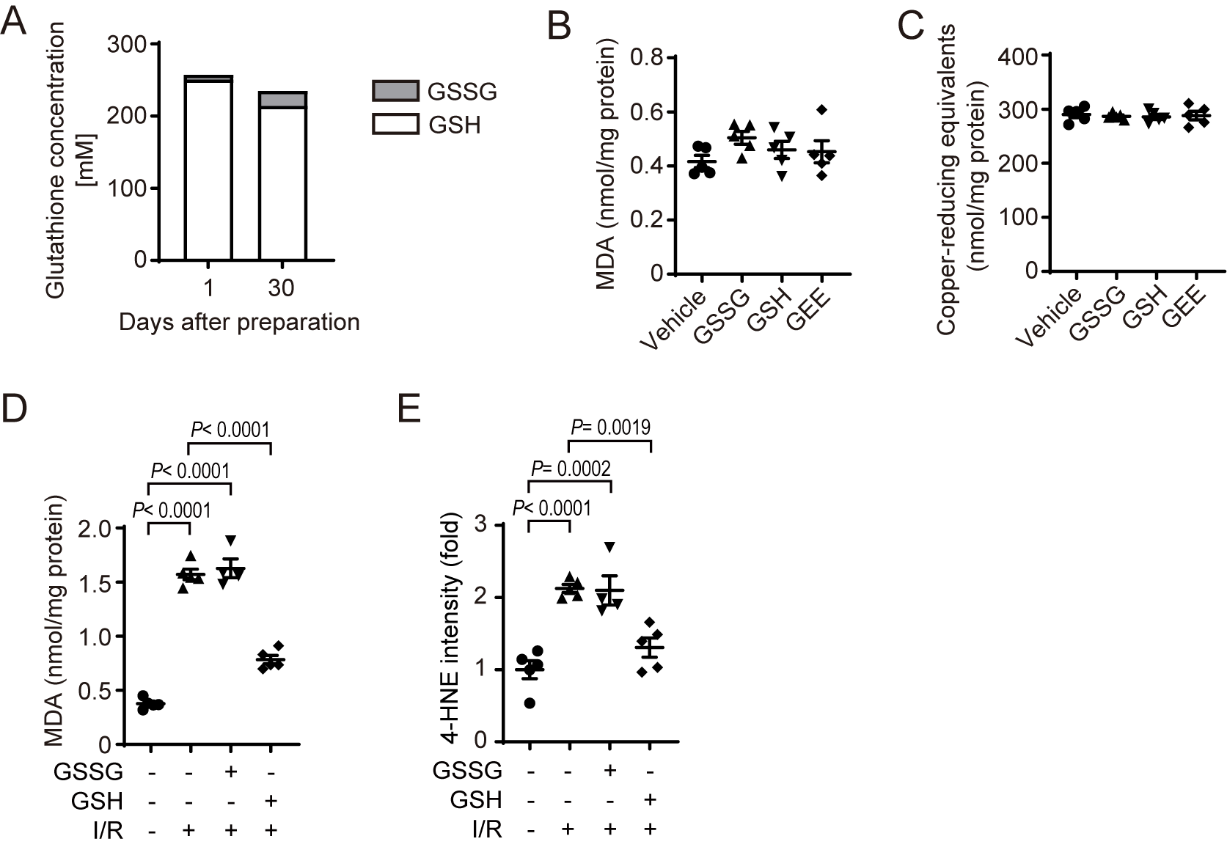


**Supplementary Fig. 11. Effect of glutathione administration on redox status of the heart.** (**A**) Stability of prepared GSH. GSH solution for the osmotic pump in Fig. 4D-M was incubated at 37 ^o^C and recovered at 1 or 30 days after incubation. GSH and GSSG concentrations were measured. (**B**, **C**) Redox status of the heart from mice infused with saline, GSSG, GSH and GEE. Malondialdehyde (MDA) for oxidative stress index (**B**) and copper-reducing equivalents for total antioxidant capacity (**C**) were measured. (n=5 mice per treatment). (**D, E**) Effect of GSSG and GSH administration on oxidative stress in the heart 1 day after ischemia/reperfusion (I/R). GSSG or GSH was infused into mice 3 days before I/R operation. Oxidative stress was evaluated by measuring MDA production (**D**) and 4-HNE intensity (**E**) (n=4 mice for I/R GSSG, n=5 mice for others). Data are shown as the means ± SEM. Significance was determined by one-way ANOVA followed by Tukey’s post-hoc test. Source data are provided as a Source Data file.


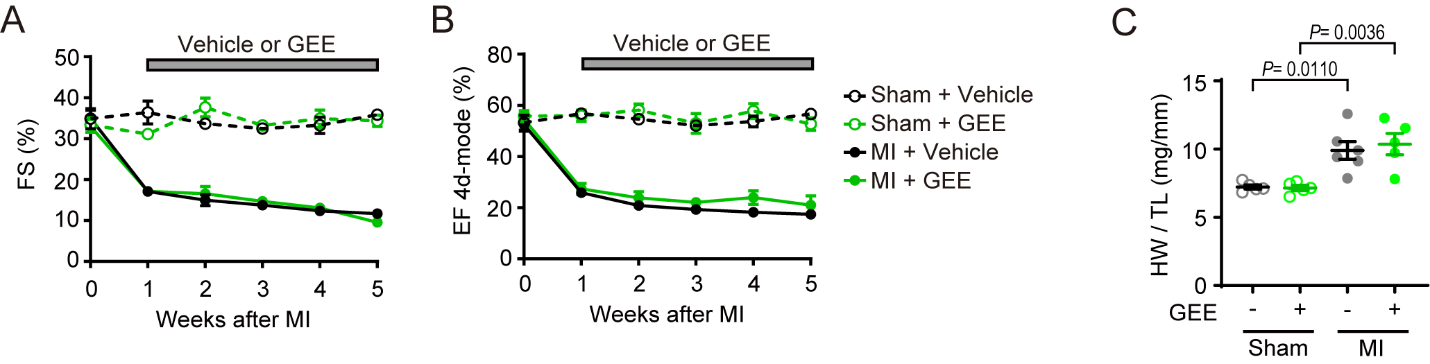


**Supplementary Fig. 12. Effect of GEE administration for MI-operated mice.** (**A**, **B**) Changes in fractional shortening (FS) (**A**) and ejection fraction (EF) (**B**) in mice after MI. An osmotic pump filled with saline (vehicle) or GEE (30 mg/kg/day) was implanted intraperitoneally at 7 days after MI. (n=6 mice for MI + vehicle, n=5 for others). (**C**) Effect of GEE on heart weight (HW) / tibia length (TL) ratio in mice 5 weeks after MI. (n=6 mice for MI + vehicle, n=5 for others). Data are shown as the means ± SEM. Significance was determined by one-way ANOVA followed by Tukey’s post-hoc test. Source data are provided as a Source Data file.

**
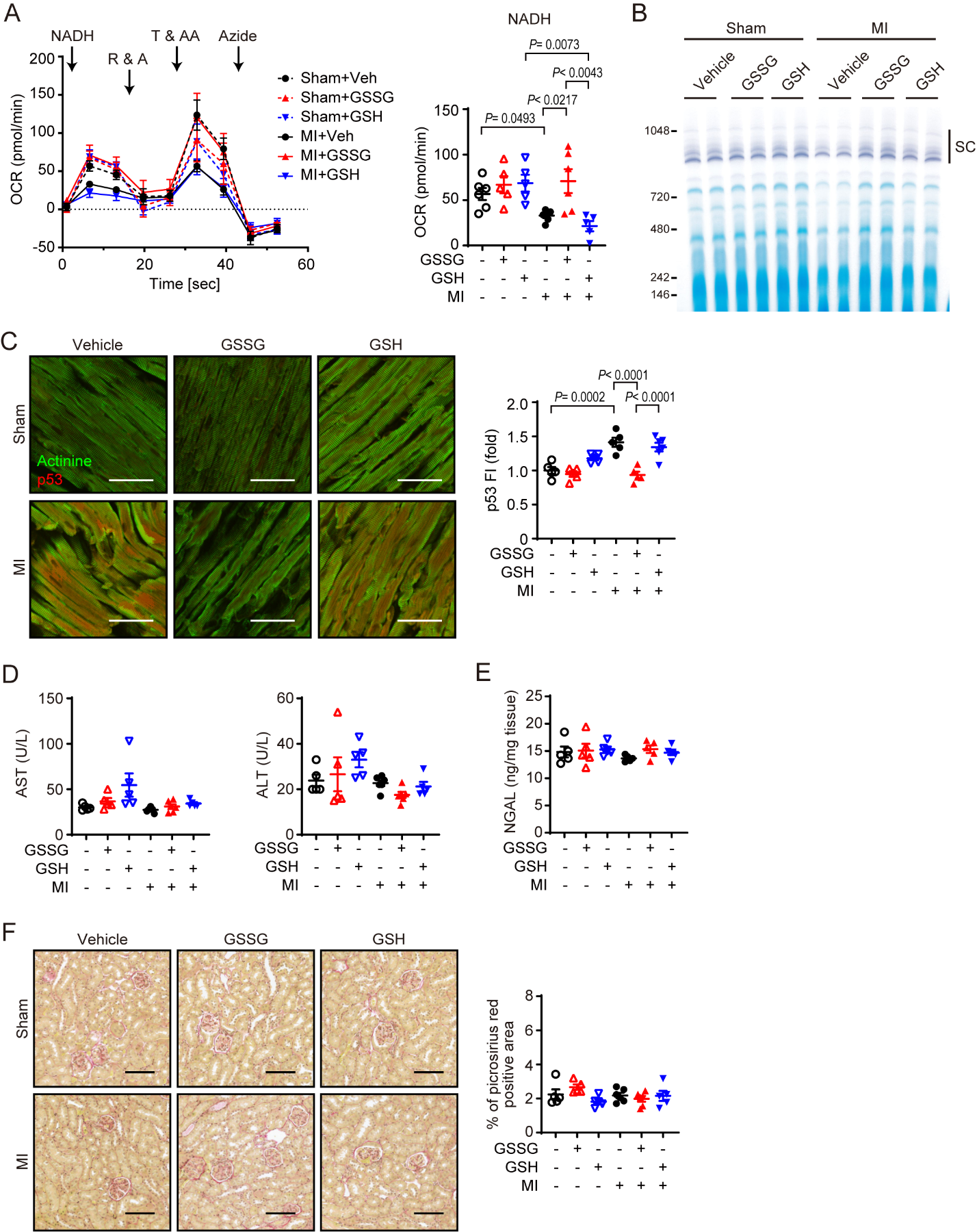
**

**Supplementary Fig. 13. Effect of glutathione administration for MI-operated mice.** (**A**) Representative seahorse profiles using RIFS respirometry protocol in frozen heart homogenates from MI-operated mice. NADH, rotenone + antimycin A (R & A), TMPD + ascorbic acid (T & AA) and azide were added at the indicated timing. Right graph shows the changes in OCR after NADH administration. (n=6 mice for Sham + Veh, MI + Veh and MI + GSSG, n=5 for others). (**B**) Representative BN-PAGE showing complex I activity (dark blue) in mitochondrial supercomplex (SC) from MI-operated mice (for Fig. 4H). (n=5 mice per treatment). (**C**) Effect of GSSG or GSH on p53 intensity in peri-infarct zone myocardium 5 weeks after MI. (n=6 mice for MI + GSH, n=5 for others). Scale bars, 50 µm. (**D**) Plasma AST and ALT levels measured 5 weeks after MI. (n=5 mice per treatment). (**E**) Kidney NGAL expression levels measured 5 weeks after MI. (n=5 mice per treatment). (**F**) Picrosirius red-stained images of kidney 5 weeks after MI. (n=5 mice per treatment). Scale bar, 100 µm. Picrosirius red-positive areas were quantified. Data are shown as the means ± SEM. Significance was determined by one-way ANOVA followed by Newman-Keuls (**A**) and Tukey’s (**C**-**F**) post-hoc test. Source data are provided as a Source Data file.


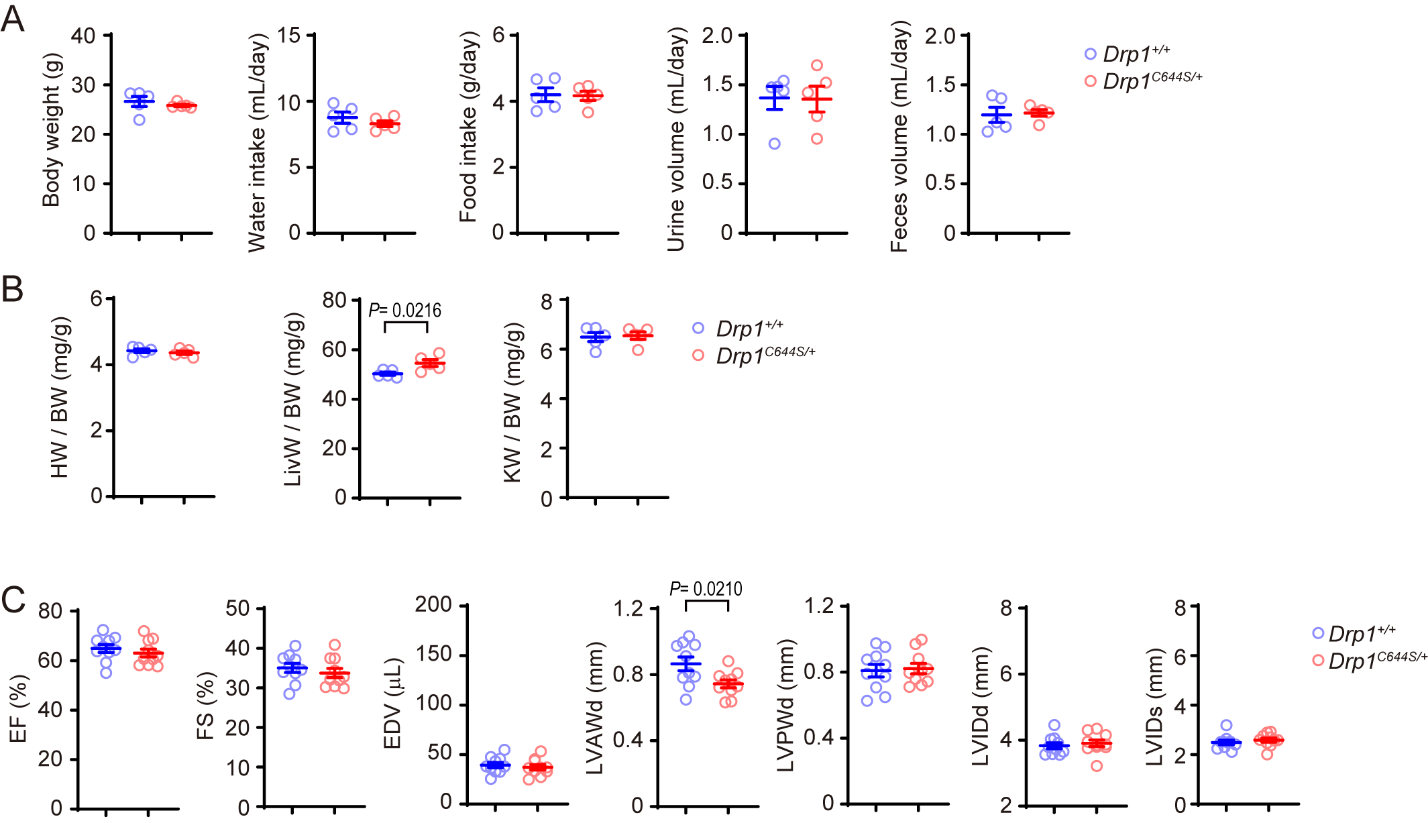


**Supplementary Fig. 14. Basal activity, organ weight and cardiac functions of Drp1^C644S/+^ hetero knock-in mouse.** (**A**) Body weight, water intake, food intake, urine volume, and feces volume in *Drp1^+/+^* and *Drp1^C644S/+^* mice. (n=5 mice per each group). (**B**) Organ weight. heart weight (HW) / body weight (BW) ratio, liver weight (LivW) / BW ratio and kidney weight (KW) / BW ratio in *Drp1^+/+^* and *Drp1^C644S/+^* mice. (n=5 mice per each group). (**C**) Cardiac functions. Ejection fraction (EF), fractional shortening (FS), end-diastolic volume (EDV), left ventricular anterior wall diameter (LVAWd), left ventricular posterior wall in diastole (LVPWd), left ventricular internal dimension in diastole (LVIDd), left ventricular internal dimension in systole (LVIDs) in *Drp1^+/+^* and *Drp1^C644S/+^* mice. (n=10 mice per each group). Data are shown as the means ± SEM. Significance was determined by two-sided unpaired t-test. Source data are provided as a Source Data file.


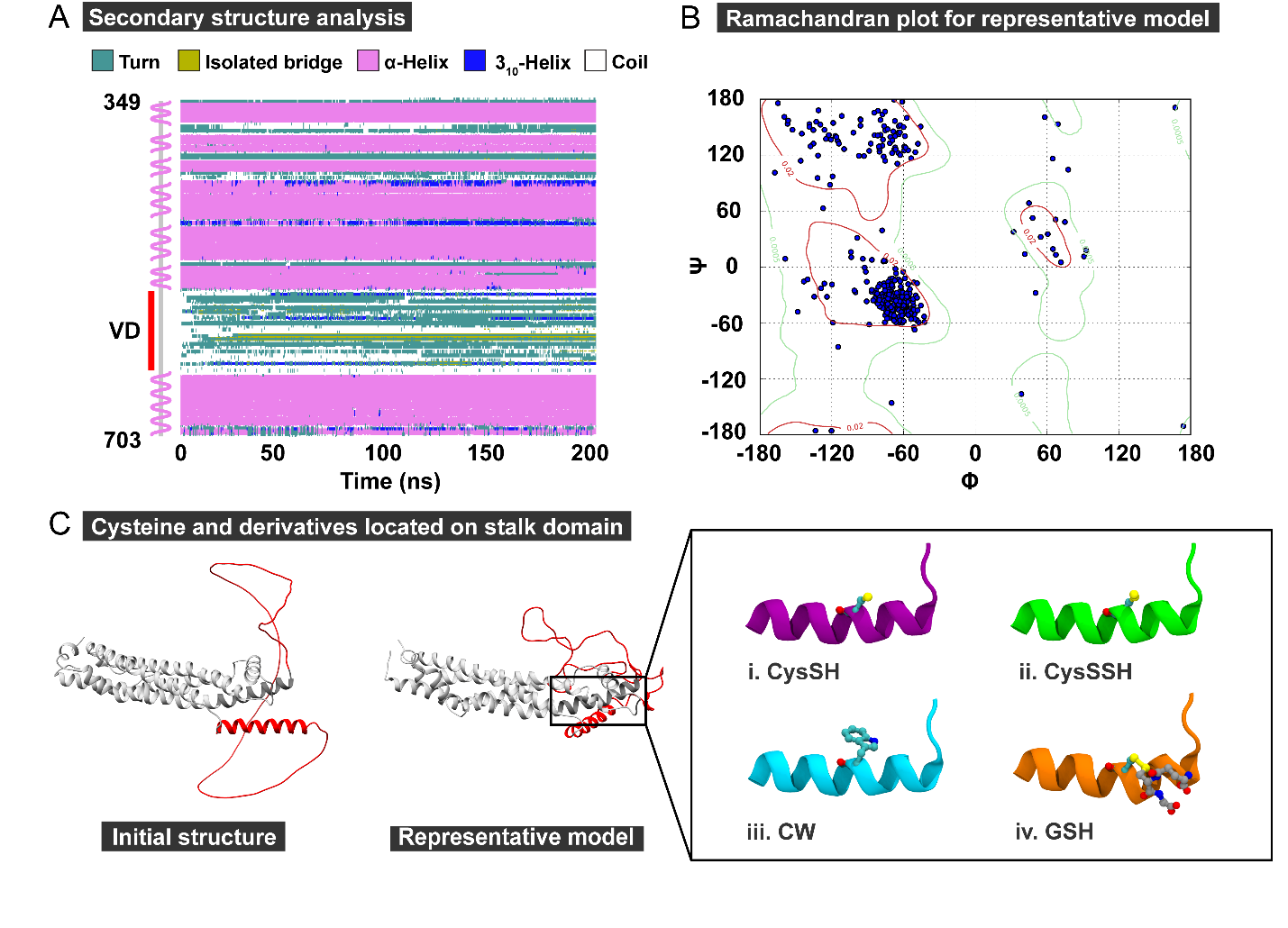


**Supplementary Fig. 15. Structural modeling and validation of Drp1 at the stalk and variable domain (VD).** (**A**) Per-residue secondary structure map during 200 ns-trajectory of Drp1 structure predicted by AlphaFold2. The representative model was obtained from RMSD clustering, and the initial structure is shown as the cartoon model. The IDR and focused stalk region of Drp1 illustrate red and gray, respectively. (**B**) Ramachandran plot for the representative model obtained from the average structure of MD trajectory. (**C**) Cysteine (CysSH) (i.) and derivatives (ii., iii., and iv.) on stalk region of Drp1.


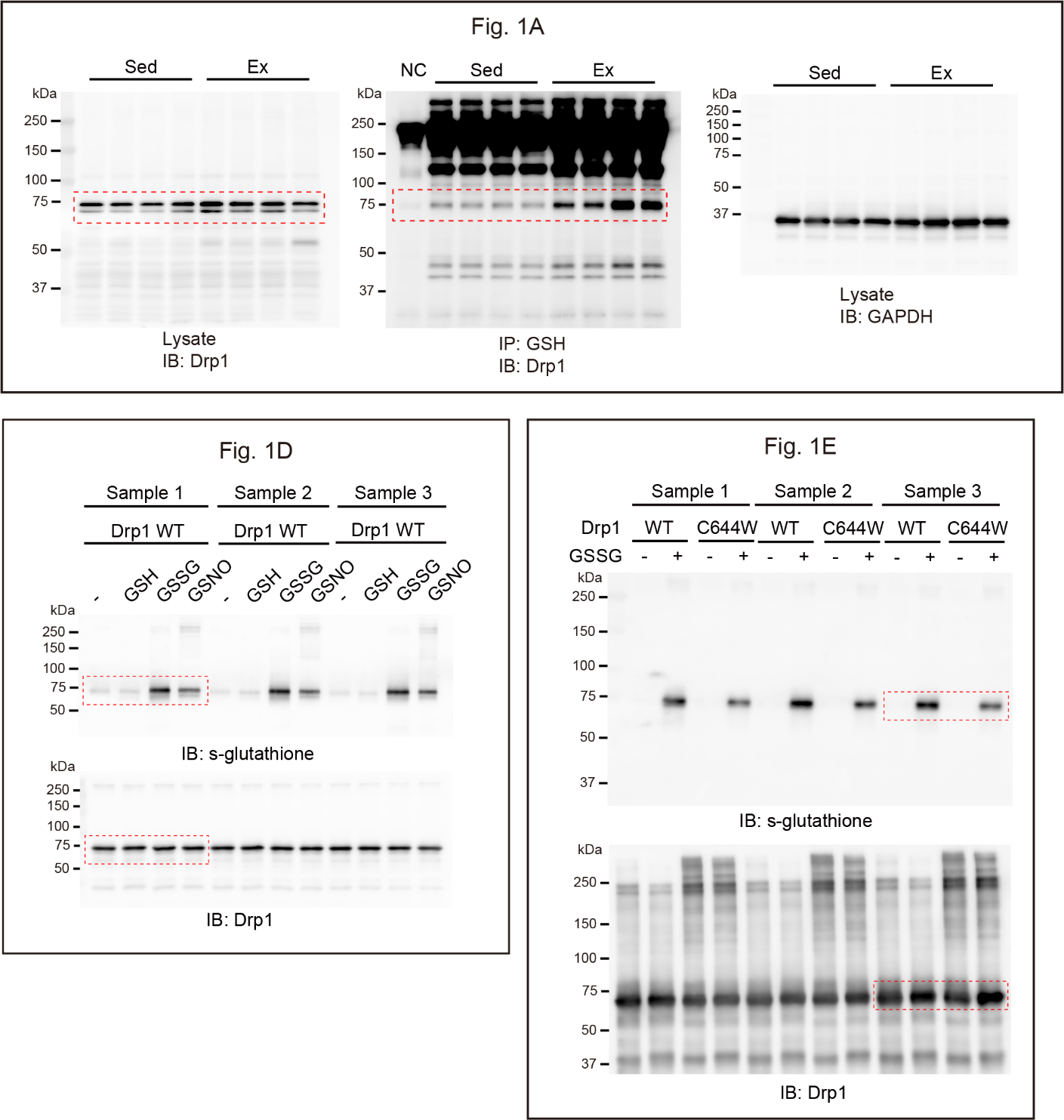


**Supplementary Fig. 16. Uncropped and multi-sample western blots with the indicated areas in Fig. 1.** Uncropped and multi-sample full western blots with molecular weight marker are shown. The dashed red boxes indicate the cropped areas presented in Fig. 1A, 1D and 1E.

**
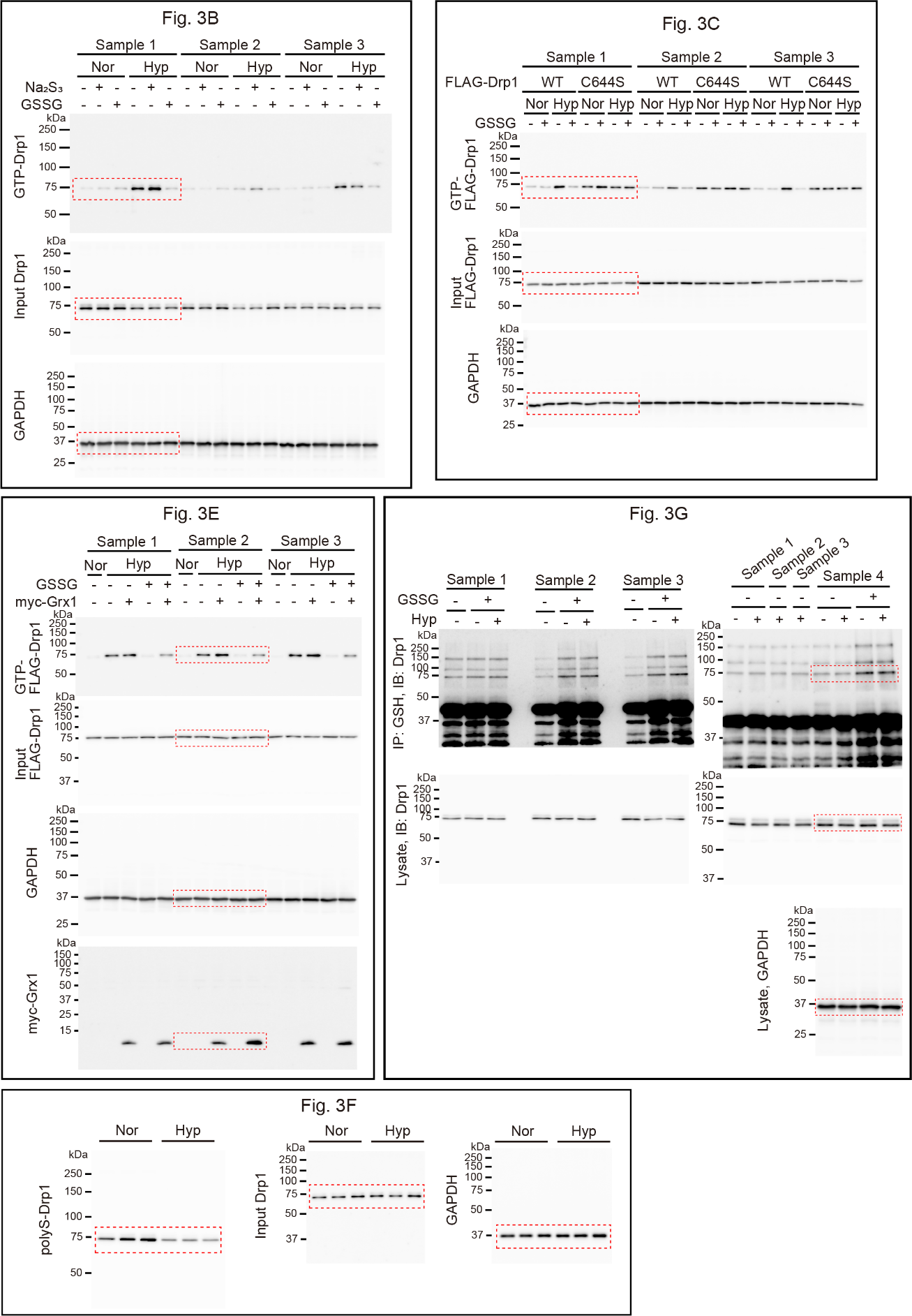
**

**Supplementary Fig. 17. Uncropped and multi-sample western blots with the indicated areas in Fig. 3.** Uncropped and multi-sample full western blots with molecular weight marker are shown. The dashed red boxes indicate the cropped areas presented in Fig. 3B, 3C, 3E, 3F and 3G.

**
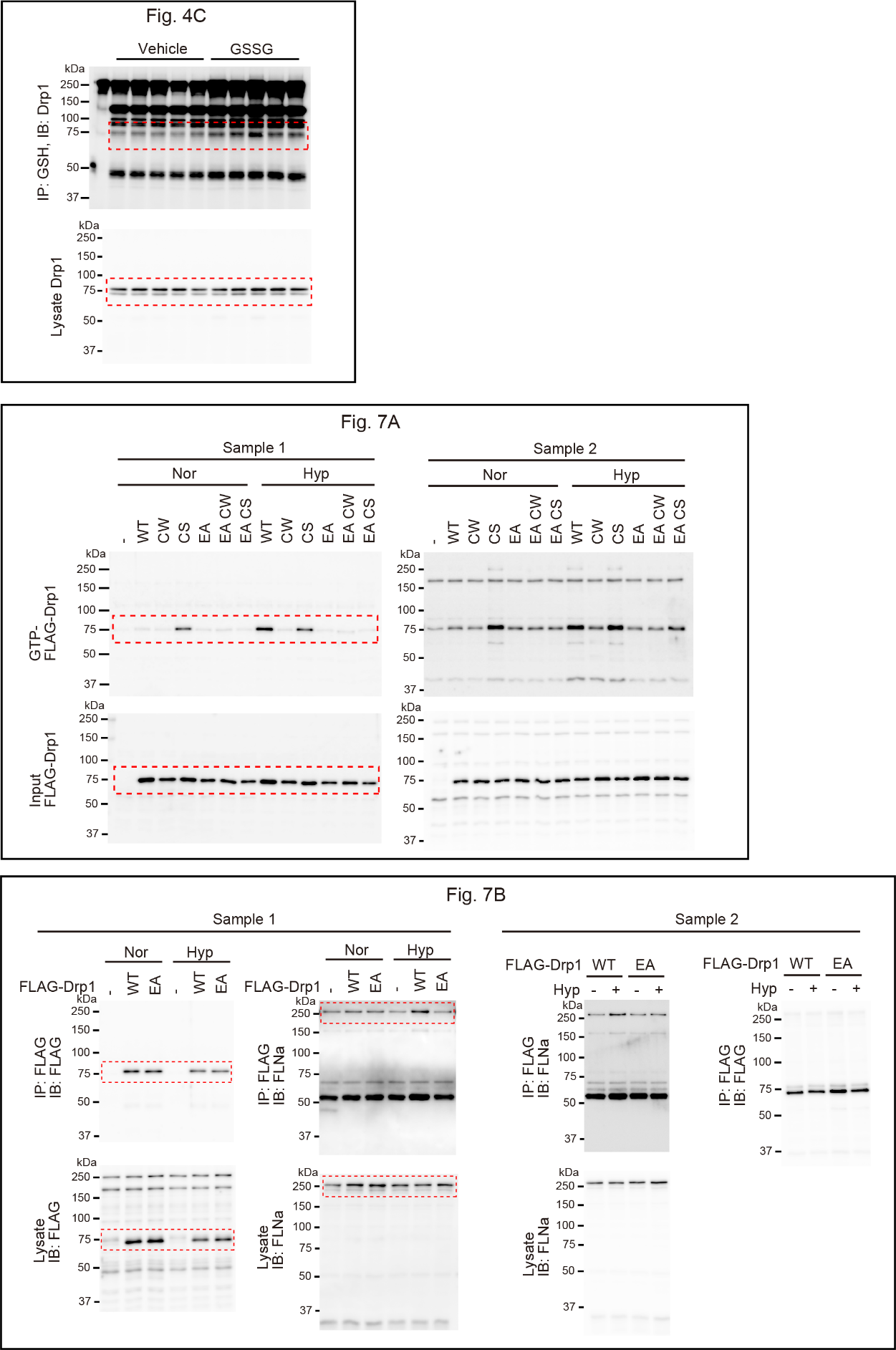
**

**Supplementary Fig. 18. Uncropped and multi-sample western blots with the indicated areas in Fig. 4 and 7.** Uncropped and multi-sample full western blots with molecular weight marker are shown. The dashed red boxes indicate the cropped areas presented in Fig. 4C, 7A and 7B.

**
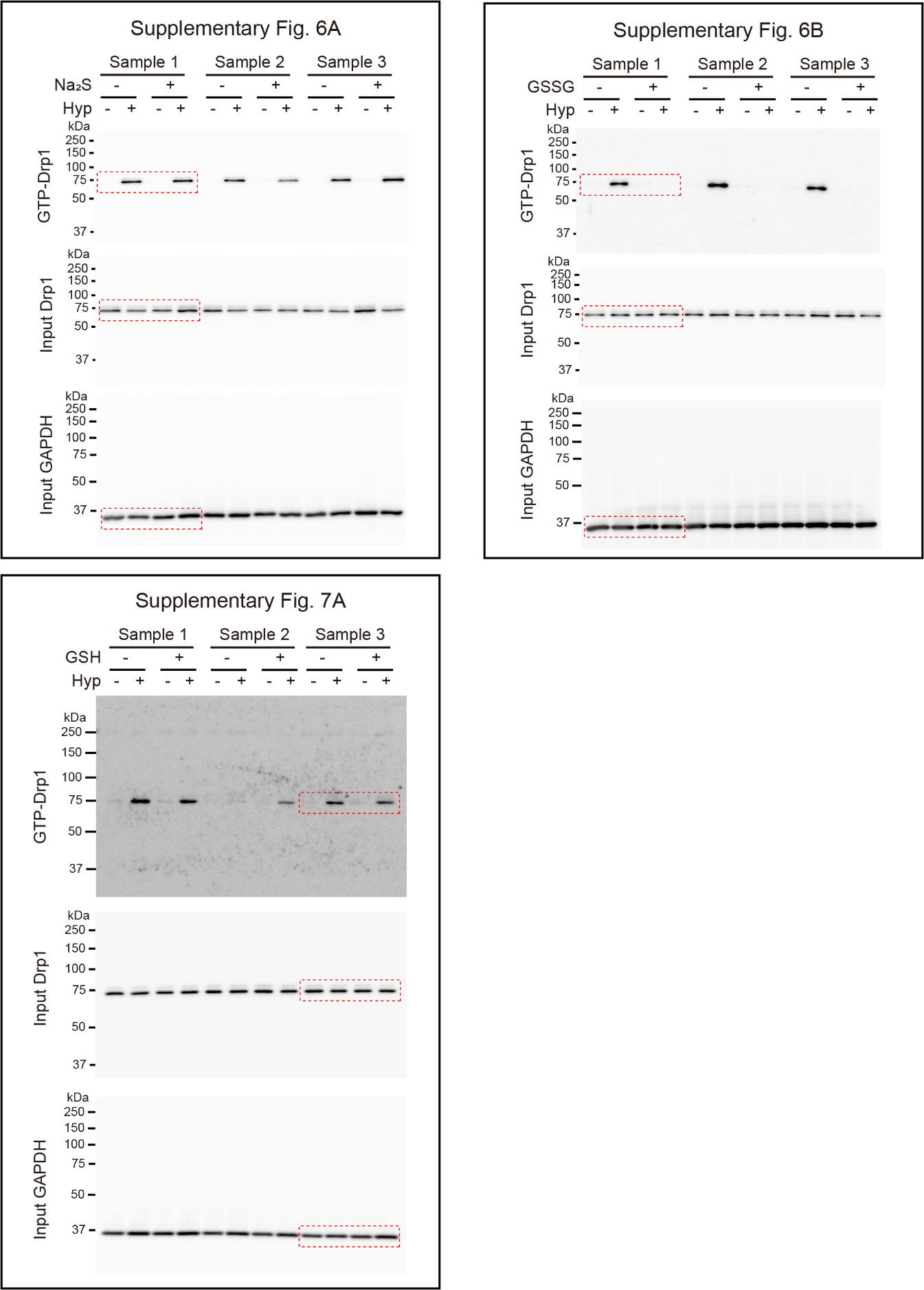
**

**Supplementary Fig. 19. Uncropped and multi-sample western blots with the indicated areas in Supplementary Fig. 6 and 7.** Uncropped and multi-sample full western blots with molecular weight marker are shown. The dashed red boxes indicate the cropped areas presented in Supplementary Fig. 6A, 6B and 7A.

**
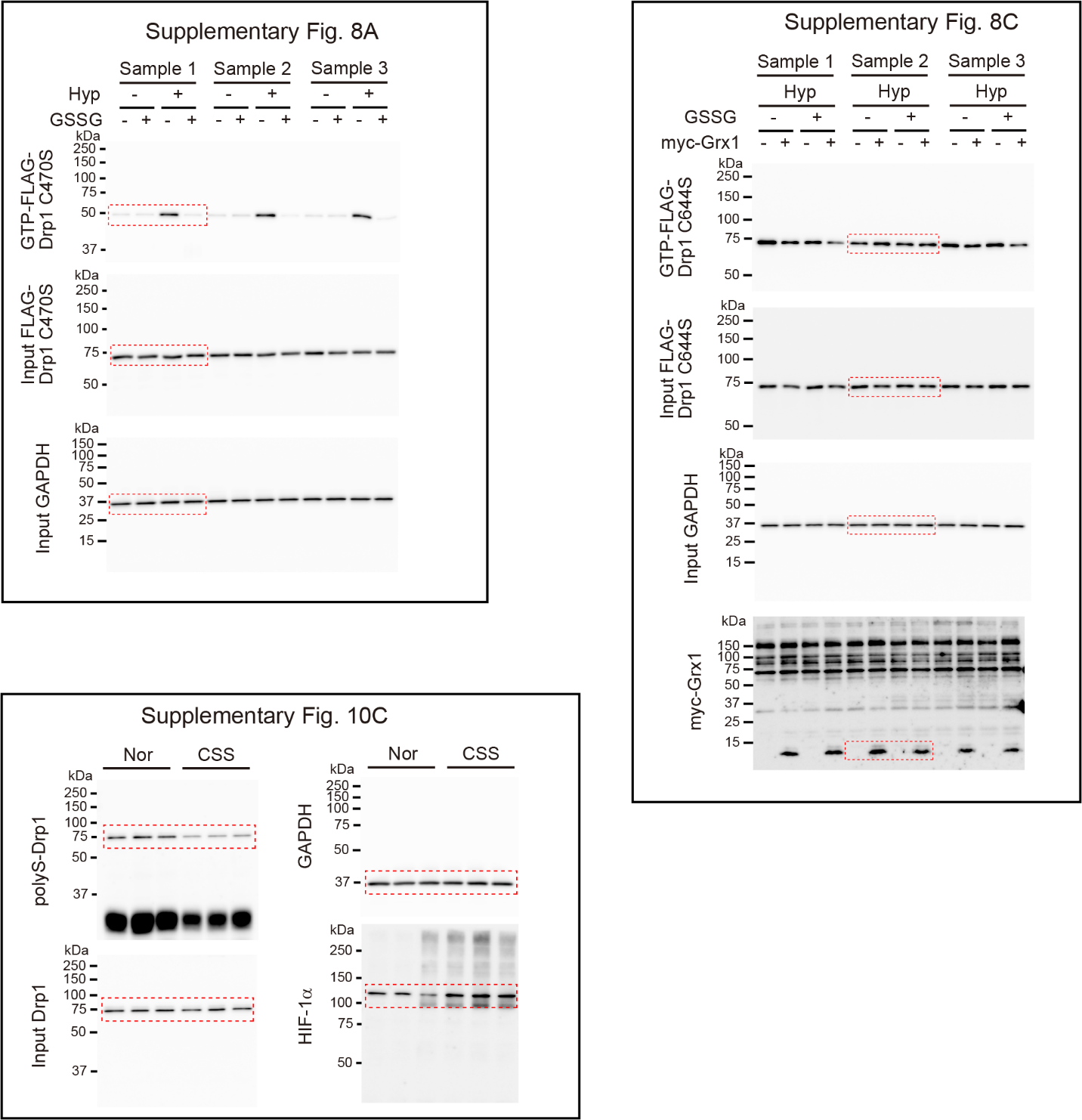
**

**Supplementary Fig. 20. Uncropped and multi-sample western blots with the indicated areas in Supplementary Fig. 8 and 10.** Uncropped and multi-sample full western blots with molecular weight marker are shown. The dashed red boxes indicate the cropped areas presented in Supplementary Fig. 8A, 8C and 10C.

Supplementary Table 1. Echocardiography parameters in GSSG and GSH groups.

|  | **Sham + Vehicle**  **(Saline)** | **Sham + GSSG**  **(30 mg/kg/day)** | **Sham + GSH**  **(30 mg/kg/day)** | **MI + Vehicle**  **(Saline)** | **MI + GSSG**  **(30 mg/kg/day)** | **MI + GSH**  **(30 mg/kg/day)** |
| --- | --- | --- | --- | --- | --- | --- |
| n | 5 | 5 | 5 | 5 | 5 | 6 |
| EDV (uL) | | | | | | |
| Week 0 | 39.5 ± 1.1 | 41.0 ± 1.1 | 40.6 ± 2.1 | 39.2 ± 2.1 | 35.9 ± 2.0 | 40.7 ± 1.9 |
| Week 1 | 33.1 ± 3.6 | 33.1 ± 0.4 | 32.2 ± 3.0 | 85.5 ± 8.2∗∗ | 68.5 ± 7.9∗ | 106.1 ± 8.8∗∗ |
| Week 2 | 35.0 ± 2.4 | 36.0 ± 2.0 | 39.8 ± 2.6 | 108.9 ± 9.1∗∗ | 84.0 ± 12.5 | 124.7 ± 10.9∗∗ |
| Week 3 | 37.5 ± 1.7 | 36.6 ± 2.9 | 37.1 ± 1.1 | 113.3 ± 10.8∗∗ | 86.8 ± 14.9 | 129.3 ± 12.5∗∗ |
| Week 4 | 37.2 ± 2.8 | 34.6 ± 1.9 | 35.4 ± 2.1 | 122.3 ± 14.7∗ | 91.7 ± 15.1 | 135.7 ± 17.1∗∗ |
| Week 5 | 36.8 ± 1.7 | 38.3 ± 2.7 | 34.0 ± 2.5 | 123.6 ± 13.9∗ | 84.4 ± 16.6 | 147.2 ± 19.9∗ |
| HR (rpm) | | | | | | |
| Week 0 | 465 ± 18 | 432 ± 2 | 431 ± 10 | 442 ± 22 | 490 ± 17 | 410 ± 25 |
| Week 1 | 443 ± 18 | 473 ± 34 | 492 ± 24 | 506 ± 22 | 420 ± 19 | 463 ± 37 |
| Week 2 | 499 ± 27 | 487 ± 14 | 476 ± 16 | 511 ± 24 | 432 ± 19 | 476 ± 20 |
| Week 3 | 493 ± 20 | 487 ± 26 | 463 ± 30 | 514 ± 18 | 468 ± 18 | 484 ± 17 |
| Week 4 | 502 ± 42 | 432 ± 33 | 475 ± 28 | 506 ± 29 | 479 ± 29 | 476 ± 30 |
| Week 5 | 489 ± 19 | 460 ± 34 | 488 ± 11 | 458 ± 31 | 514 ± 30 | 506 ± 17 |
| LVAWd (mm) | | | | | | |
| Week 0 | 0.78 ± 0.04 | 0.84 ± 0.03 | 0.79 ± 0.02 | 0.72 ± 0.05 | 0.78 ± 0.02 | 0.83 ± 0.04 |
| Week 1 | 0.90 ± 0.04 | 0.82 ± 0.05 | 0.96 ± 0.09 | 0.80 ± 0.06 | 0.64 ± 0.04∗∗ | 0.81 ± 0.06 |
| Week 2 | 0.98 ± 0.1.0 | 0.89 ± 0.05 | 0.94 ± 0.05 | 0.62 ± 0.03 | 0.62 ± 0.03 | 0.71 ± 0.10 |
| Week 3 | 0.74 ± 0.09 | 0.83 ± 0.02 | 0.93 ± 0.04 | 0.68 ± 0.10 | 0.83 ± 0.03 | 0.66 ± 0.04 |
| Week 4 | 0.84 ± 0.06 | 0.77 ± 0.03 | 0.95 ± 0.06 | 0.63 ± 0.06 | 0.60 ± 0.05 | 0.72 ± 0.10 |
| Week 5 | 0.80 ± 0.05 | 0.83 ± 0.07 | 0.92 ± 0.04 | 0.58 ± 0.09 | 0.62 ± 0.07 | 0.74 ± 0.11 |
| LVPWd (mm) | | | | | | |
| Week 0 | 0.79 ± 0.02 | 0.90 ± 0.07 | 0.87 ± 0.04 | 0.71 ± 0.03 | 0.86 ± 0.09 | 0.90 ± 0.04# |
| Week 1 | 0.80 ± 0.05 | 0.90 ± 0.06 | 0.88 ± 0.07 | 0.83 ± 0.09 | 0.99 ± 0.09 | 1.03 ± 0.05 |
| Week 2 | 0.98 ± 0.05 | 0.99 ± 0.07 | 0.73 ± 0.04∗ | 0.79 ± 0.06 | 0.89 ± 0.05 | 0.97 ± 0.07 |
| Week 3 | 0.89 ± 0.07 | 0.90 ± 0.05 | 0.86 ± 0.08 | 0.87 ± 0.07 | 0.81 ± 0.05 | 1.01 ± 0.04 |
| Week 4 | 0.93 ± 0.04 | 0.88 ± 0.07 | 0.83 ± 0.05 | 0.78 ± 0.07 | 0.92 ± 0.05 | 1.06 ± 0.06 |
| Week 5 | 0.96 ± 0.07 | 0.93 ± 0.04 | 0.81 ± 0.04 | 0.72 ± 0.10 | 0.95 ± 0.06 | 1.04 ± 0.04 |
| LVIDd (mm) | | | | | | |
| Week 0 | 3.91 ± 0.07 | 3.92 ± 0.04 | 3.72 ± 0.10 | 4.04 ± 0.15 | 3.69 ± 0.10 | 3.75 ± 0.08 |
| Week 1 | 3.53 ± 0.19 | 3.76 ± 0.07 | 3.61 ± 0.15 | 4.98 ± 0.23∗∗ | 4.87 ± 0.08∗∗ | 4.84 ± 0.09∗∗ |
| Week 2 | 3.91 ± 0.26 | 3.75 ± 0.08 | 3.74 ± 0.12 | 5.64 ± 0.12∗∗ | 5.08 ± 0.19∗ | 5.39 ± 0.21∗ |
| Week 3 | 3.74 ± 0.05 | 3.75 ± 0.12 | 3.70 ± 0.12 | 5.65 ± 0.15∗∗ | 5.07 ± 0.25∗ | 5.52 ± 0.27∗∗ |
| Week 4 | 3.72 ± 0.05 | 3.73 ± 0.07 | 3.73 ± 0.09 | 5.87 ± 0.16∗∗ | 5.21 ± 0.27∗ | 5.50 ± 0.27∗∗ |
| Week 5 | 3.79 ± 0.10 | 3.95 ± 0.20 | 3.59 ± 0.19 | 5.98 ± 0.18∗∗ | 5.13 ± 0.20∗∗ | 5.72 ± 0.31∗∗ |
| LVIDs (mm) | | | | | | |
| Week 0 | 2.59 ± 0.04 | 2.59 ± 0.05 | 2.39 ± 0.13 | 2.72 ± 0.14 | 2.44 ± 0.07 | 2.48 ± 0.07 |
| Week 1 | 2.34 ± 0.15 | 2.52 ± 0.12 | 2.33 ± 0.15 | 4.10 ± 0.24∗∗ | 3.90 ± 0.09∗∗ | 3.93 ± 0.10∗∗ |
| Week 2 | 2.61 ± 0.23 | 2.42 ± 0.07 | 2.54 ± 0.13 | 4.85 ± 0.15∗∗ | 4.12 ± 0.23∗∗ | 4.57 ± 0.20∗∗ |
| Week 3 | 2.36 ± 0.06 | 2.42 ± 0.10 | 2.47 ± 0.15 | 4.91 ± 0.18∗∗ | 3.97 ± 0.24∗∗ | 4.78 ± 0.27∗∗ |
| Week 4 | 2.39 ± 0.09 | 2.34 ± 0.10 | 2.45 ± 0.13 | 5.10 ± 0.15∗∗ | 4.12 ± 0.28∗ | 4.80 ± 0.25∗∗ |
| Week 5 | 2.44 ± 0.08 | 2.65 ± 0.18 | 2.34 ± 0.21 | 5.32 ± 0.23∗∗ | 4.03 ± 0.19∗# | 5.11 ± 0.31∗∗ |

EDV: end-diastolic volume; SV: stroke volume; CO: cardiac output; HR: heart rate; LVAWd: LV anterior wall thickness at end-diastole; LVPWd: LV posterior wall thickness at end-diastole; LVIDd: LV internal diameter at end-diastole; LVIDs: LV internal diameter at end-systole. Data are shown as mean ± s.e.m. ∗P < 0.05, ∗∗P < 0.01 vs Sham + Vehicle; #P < 0.05, ##P < 0.01 vs MI + Vehicle. Significance was determined using two-way ANOVA followed by Sidak’s post-hoc test.

Supplementary Table 2. Organ weight parameters in GSSG and GSH groups.

|  | **Sham + Vehicle**  **(Saline)** | **Sham + GSSG**  **(30 mg/kg/day)** | **Sham + GSH**  **(30 mg/kg/day)** | **MI + Vehicle**  **(Saline)** | **MI + GSSG**  **(30 mg/kg/day)** | **MI + GSH**  **(30 mg/kg/day)** |
| --- | --- | --- | --- | --- | --- | --- |
| n | 5 | 5 | 5 | 5 | 5 | 6 |
| BW (g) | 26.9 ± 0.8 | 27.8 ± 0.3 | 27.7 ± 0.5 | 28.2 ± 0.6 | 26.9 ± 0.5 | 28.5 ± 0.5 |
| HW (mg) | 127 ± 4 | 121 ± 1 | 120 ± 4 | 189 ± 9∗∗ | 148 ± 9## | 194 ± 8∗∗ |
| HW/BW (mg/g) | 4.71 ± 0.13 | 4.34 ± 0.11 | 4.33 ± 0.07 | 6.68 ± 0.26∗∗ | 5.5 ± 0.27## | 6.8 ± 0.23∗∗ |
| HW/TL (g/cm) | 0.07 ± 0.002 | 0.066 ± 0.002 | 0.069 ± 0.002 | 0.107 ± 0.004∗∗ | 0.085 ± 0.005∗## | 0.111 ± 0.004∗∗ |

BW: body weight; HW: heart weight; TL: tibia length. Data are shown as mean ± s.e.m. ∗P < 0.05, ∗∗P < 0.01 vs Sham + Vehicle. #P < 0.05, ##P < 0.01 vs MI + Vehicle. Significance was determined using one-way ANOVA followed by Tukey’s post-hoc test.

Supplementary Table 3. Echocardiography parameters in GEE groups.

|  | **Sham + Vehicle**  **(Saline)** | **Sham + GEE**  **(30 mg/kg/day)** | **MI + Vehicle**  **(Saline)** | **MI + GEE**  **(30 mg/kg/day)** |
| --- | --- | --- | --- | --- |
| n | 5 | 5 | 6 | 5 |
| EDV (uL) | | | | |
| Week 0 | 31.7 ± 1.7 | 42.2 ± 3.2 | 32.8 ± 1.7 | 38.7 ± 1.8 |
| Week 1 | 32.2 ± 1.7 | 39.9 ± 3.0 | 85.7 ± 7.2** | 81.5 ± 7.5** |
| Week 2 | 31.6 ± 1.3 | 35.3 ± 2.8 | 99.2 ± 10.2** | 92.5 ± 9.6** |
| Week 3 | 32.2 ± 2.1 | 37.5 ± 1.2 | 98.3 ± 8.1** | 93.9 ± 8.2** |
| Week 4 | 29.7 ± 1.2 | 38.8 ± 2.4 | 98.7 ± 9.4** | 97.9 ± 9.7** |
| Week 5 | 31.8 ± 1.1 | 33.7 ± 2.0 | 103.0 ± 9.0** | 97.0 ± 10.2** |
| EF (%) 4D-Mode | | | | |
| Week 0 | 53.2 ± 2.9 | 55.8 ± 2.2 | 52.4 ± 2.5 | 54.6 ± 2.2 |
| Week 1 | 56.7 ± 1.4 | 55.9 ± 2.2 | 25.8 ± 1.7** | 27.4 ± 2.2** |
| Week 2 | 54.5 ± 1.3 | 58.1 ± 2.4 | 20.8 ± 1.4** | 23.8 ± 2.5** |
| Week 3 | 52.0 ± 1.4 | 52.9 ± 3.9 | 19.3 ± 1.3** | 22.0 ± 1.8** |
| Week 4 | 53.7 ± 2.1 | 57.7 ± 2.9 | 18.2 ± 1.7** | 23.9 ± 2.7** |
| Week 5 | 56.6 ± 1.8 | 52.7 ± 2.4 | 17.4 ± 0.7** | 21.0 ± 3.7** |
| HR (rpm) | | | | |
| Week 0 | 451 ± 26 | 492 ± 14 | 450 ± 21 | 434 ± 32 |
| Week 1 | 512 ± 20 | 465 ± 19 | 508 ± 15 | 497 ± 19 |
| Week 2 | 465 ± 24 | 502 ± 17 | 485 ± 15 | 455 ± 20 |
| Week 3 | 467 ± 23 | 498 ± 27 | 456 ± 22 | 441 ± 19 |
| Week 4 | 498 ± 22 | 473 ± 23 | 481 ± 43 | 515 ± 23 |
| Week 5 | 465 ± 21 | 523 ± 24 | 450 ± 23 | 423 ± 16 |
| LVAWd (mm) | | | | |
| Week 0 | 0.66 ± 0.03 | 0.81 ± 0.03* | 0.66 ± 0.02 | 0.81 ± 0.03*†† |
| Week 1 | 0.78 ± 0.04 | 0.80 ± 0.05 | 0.61 ± 0.02* | 0.70 ± 0.05 |
| Week 2 | 0.75 ± 0.05 | 0.79 ± 0.04 | 0.49 ± 0.05* | 0.63 ± 0.10 |
| Week 3 | 0.89 ± 0.05 | 0.87 ± 0.07 | 0.50 ± 0.04** | 0.48 ± 0.03** |
| Week 4 | 0.85 ± 0.03 | 0.88 ± 0.02 | 0.50 ± 0.05** | 0.52 ± 0.02** |
| Week 5 | 0.87 ± 0.03 | 0.91 ± 0.05 | 0.49 ± 0.03** | 0.43 ± 0.05** |
| LVPWd (mm) | | | | |
| Week 0 | 0.71 ± 0.05 | 0.79 ± 0.03 | 0.70 ± 0.04 | 0.93 ± 0.01* |
| Week 1 | 0.78 ± 0.05 | 0.85 ± 0.10 | 0.94 ± 0.06 | 0.91 ± 0.11 |
| Week 2 | 0.82 ± 0.05 | 0.80 ± 0.05 | 0.95 ± 0.04 | 0.86 ± 0.06 |
| Week 3 | 0.70 ± 0.05 | 0.80 ± 0.05 | 0.97 ± 0.06* | 0.92 ± 0.04* |
| Week 4 | 0.73 ± 0.05 | 0.77 ± 0.03 | 0.87 ± 0.05 | 0.87 ± 0.08 |
| Week 5 | 0.83 ± 0.08 | 0.80 ± 0.07 | 0.86 ± 0.06 | 0.89 ± 0.07 |
| LVIDd (mm) | | | | |
| Week 0 | 3.67 ± 0.11 | 3.90 ± 0.10 | 3.71 ± 0.10 | 3.95 ± 0.08 |
| Week 1 | 3.77 ± 0.10 | 3.96 ± 0.16 | 5.26 ± 0.21** | 4.83 ± 0.19* |
| Week 2 | 3.78 ± 0.12 | 3.82 ± 0.12 | 5.48 ± 0.17** | 5.22 ± 0.21* |
| Week 3 | 3.74 ± 0.22 | 3.98 ± 0.08 | 5.54 ± 0.13** | 5.35 ± 0.23* |
| Week 4 | 3.83 ± 0.10 | 3.86 ± 0.09 | 5.62 ± 0.19** | 5.54 ± 0.18** |
| Week 5 | 3.83 ± 0.13 | 3.84 ± 0.11 | 5.69 ± 0.19** | 5.60 ± 0.31* |
| LVIDs (mm) | | | | |
| Week 0 | 2.40 ± 0.15 | 2.60 ± 0.10 | 2.42 ± 0.12 | 2.65 ± 0.07 |
| Week 1 | 2.40 ± 0.13 | 2.73 ± 0.14 | 4.37 ± 0.21** | 4.01 ± 0.19** |
| Week 2 | 2.51 ± 0.13 | 2.39 ± 0.15 | 4.67 ± 0.20** | 4.37 ± 0.25* |
| Week 3 | 2.53 ± 0.17 | 2.66 ± 0.08 | 4.79 ± 0.15** | 4.57 ± 0.23** |
| Week 4 | 2.56 ± 0.13 | 2.52 ± 0.11 | 4.93 ± 0.19** | 4.82 ± 0.16** |
| Week 5 | 2.46 ± 0.11 | 2.52 ± 0.11 | 5.03 ± 0.20** | 5.07 ± 0.32* |
| EF (%) M-Mode | | | | |
| Week 0 | 64.7 ± 3.4 | 62.5 ± 2.5 | 64.8 ± 2.8 | 62.0 ± 1.6 |
| Week 1 | 66.5 ± 3.5 | 59.4 ± 1.8 | 35.4 ± 1.8** | 35.7 ± 2.0** |
| Week 2 | 63.1 ± 1.9 | 68.1 ± 3.1 | 31.3 ± 2.6** | 34.3 ± 3.3** |
| Week 3 | 61.6 ± 1.4 | 62.4 ± 1.5 | 29.0 ± 1.5** | 30.8 ± 2.0** |
| Week 4 | 62.4 ± 2.8 | 64.5 ± 2.9 | 26.3 ± 1.4** | 27.6 ± 1.5** |
| Week 5 | 66.1 ± 1.3 | 64 ± 2.0 | 24.9 ± 1.4** | 20.7 ± 2.0** |
| FS (%) | | | | |
| Week 0 | 34.9 ± 2.5 | 33.4 ± 1.8 | 35.0 ± 2.0 | 33.0 ± 1.1 |
| Week 1 | 36.4 ± 2.8 | 31.1 ± 1.2 | 17.1 ± 1.0* | 17.1 ± 1.1* |
| Week 2 | 33.6 ± 1.3 | 37.6 ± 2.3 | 15.0 ± 1.4** | 16.5 ± 1.8** |
| Week 3 | 32.5 ± 0.9 | 33.2 ± 1.1 | 13.7 ± 0.7** | 14.7 ± 1.0** |
| Week 4 | 33.3 ± 2.0 | 34.8 ± 2.1 | 12.3 ± 0.7** | 13.0 ± 0.8** |
| Week 5 | 35.8 ± 0.9 | 34.4 ± 1.4 | 11.7 ± 0.7** | 9.6 ± 0.9** |

EDV: end diastolic volume; SV: stroke volume; EF: ejection fraction; CO: cardiac output; HR: heart rate; LVAWd: LV anterior wall thickness at end diastole; LVPWd: LV posterior wall thickness at end diastole; LVIDd: LV internal diameter at end diastole; LVIDs: LV internal diameter at end systole; FS: fractional shortening. Data are shown as mean ± s.e.m. *P < 0.05, **P < 0.01 vs Sham + Vehicle. †P < 0.05, ††P < 0.01 vs MI + Vehicle. Significance was determined using two-way ANOVA followed by Sidak’s post-hoc test.

Supplementary Table 4. Organ weight parameters in GEE groups.

|  | **Sham + Vehicle**  **(Saline)** | **Sham + GEE**  **(30 mg/kg/day)** | **MI + Vehicle**  **(Saline)** | **MI + GEE**  **(30 mg/kg/day)** |
| --- | --- | --- | --- | --- |
| n | 5 | 5 | 6 | 5 |
| BW (g) | 28.5 ± 0.8 | 28.8 ± 0.7 | 29.2 ± 0.4 | 30.5 ± 0.9 |
| HW (mg) | 126 ± 2 | 127 ± 5 | 174 ± 11** | 181 ± 14** |
| HW/BW (mg/g) | 4.45 ± 0.16 | 4.39 ± 0.11 | 5.95 ± 0.35** | 5.92 ± 0.34** |
| HW/TL (g/cm) | 0.072 ± 0.002 | 0.071 ± 0.002 | 0.099 ± 0.006** | 0.104 ± 0.008** |
| LivW/BW (mg/g) | 46.1 ± 2.0 | 43.4 ± 3.0 | 41.4 ± 1.0 | 44.0 ± 0.6 |
| KW/BW (mg/g) | 5.81 ± 0.16 | 6.42 ± 0.18* | 5.35 ± 0.11 | 6.14 ± 0.10†† |

BW: body weight; HW: heart weight; TL: tibia length; LivW: liver weight; KW: kidney weight. Data are shown as mean ± s.e.m. *P < 0.05, **P < 0.01 vs Sham + Vehicle. †P < 0.05, ††P < 0.01 vs MI + Vehicle. Significance was determined using two-way ANOVA followed by Tukey’s multiple comparison test.

Supplementary Table 5. Echocardiography parameters of *Drp1^+/+^* and *Drp1^C644S/+^* mice at 1 week after MI.

|  | ***Drp1^+/+^***  **Sham** | ***Drp1^+/+^***  **MI** | ***Drp1^C644S/+^***  **Sham** | ***Drp1^C644S/+^***  **MI** |
| --- | --- | --- | --- | --- |
| n | 10 | 10 | 10 | 11 |
| EDV  (µL) | 41.5 ± 2.3 | 82.7 ± 8.4** | 36.9 ± 2.0 | 110 ± 7.4**† |
| EF (%)  4D-Mode | 56.0 ± 1.7 | 25.5 ± 1.1** | 51.7 ± 1.3 | 18.0 ± 1.9**†† |
| HR  (rpm) | 519 ± 10 | 500 ± 11 | 469 ± 18 | 502 ± 12 |
| LVAWd  (mm) | 0.89 ± 0.03 | 0.58 ± 0.02** | 0.75 ± 0.02 | 0.63 ± 0.03** |
| LVPWd  (mm) | 0.80 ± 0.04 | 0.97 ± 0.04* | 0.77 ± 0.02 | 0.92 ± 0.06 |
|  | | | | |
| LVIDd  (mm) | 3.93 ± 0.12 | 5.03 ± 0.16** | 3.81 ± 0.08 | 5.62 ± 0.13**†† |
| LVIDs  (mm) | 2.56 ± 0.10 | 4.16 ± 0.18** | 2.58 ± 0.10 | 4.96 ± 0.15**†† |
| EF (%)  M-Mode | 64.9 ± 1.5 | 36.1 ± 2.3** | 61.4 ± 2.0 | 25.3 ± 1.9**†† |
| FS  (%) | 35.1 ± 1.1 | 17.5 ± 1.2** | 32.5 ± 1.4 | 11.9 ± 1.0**†† |

EDV: end diastolic volume; EF: ejection fraction; HR: heart rate; LVAWd: LV anterior wall thickness at end diastole; LVPWd: LV posterior wall thickness at end diastole; LVIDd: LV internal diameter at end diastole; LVIDs: LV internal diameter at end systole; FS: fractional shortening. Data are shown as mean ± s.e.m. *P < 0.05, **P < 0.01 vs Vehicle within each strain. †P < 0.05, ††P < 0.01 vs *Drp1^+/+^* + MI. Significance was determined using two-way ANOVA followed by Tukey’s multiple comparison test.

Supplementary Table 6. Echocardiography parameters of *Drp1^+/+^* and *Drp1^C644S/+^* mice at 4 weeks after MI.

|  | ***Drp1^+/+^***  **Sham + Vehicle**  **(Saline)** | ***Drp1^+/+^***  **Sham + GSSG**  **(30 mg/kg/day)** | ***Drp1^C644S/+^***  **Sham + Vehicle**  **(Saline)** | ***Drp1^C644S/+^***  **Sham + GSSG**  **(30 mg/kg/day)** |
| --- | --- | --- | --- | --- |
| n | 5 | 5 | 5 | 5 |
| EDV  (µL) | 37.2 ± 2.8 | 34.6 ± 1.9 | 35.4 ± 2.1 | 38.8 ± 2.4 |
| EF (%)  4D-Mode | 56.6 ± 2.3 | 55.1 ± 1.2 | 53.1 ± 1.6 | 54.8 ± 2.3 |
| HR  (rpm) | 519 ± 23 | 519 ± 22 | 506 ± 17 | 503 ± 16 |
| LVAWd  (mm) | 0.89 ± 0.05 | 0.86 ± 0.02 | 0.80 ± 0.04 | 0.83 ± 0.04 |
| LVPWd  (mm) | 0.93 ± 0.07 | 0.70 ± 0.02 | 0.76 ± 0.08 | 0.77 ± 0.06 |
|  | | | | |
| LVIDd  (mm) | 4.15 ± 0.25 | 3.97 ± 0.15 | 3.91 ± 0.09 | 3.97 ± 0.14 |
| LVIDs  (mm) | 2.80 ± 0.21 | 2.69 ± 0.13 | 2.65 ± 0.08 | 2.68 ± 0.15 |
| EF (%)  M-Mode | 61.2 ± 1.4 | 61.7 ± 2.3 | 61.7 ± 2.1 | 61.1 ± 2.2 |
| FS  (%) | 32.9 ± 1.4 | 32.4 ± 1.5 | 32.4 ± 1.0 | 32.8 ± 1.5 |

|  | ***Drp1^+/+^***  **MI + Vehicle**  **(Saline)** | ***Drp1^+/+^***  **MI + GSSG**  **(30 mg/kg/day)** | ***Drp1^C644S/+^***  **MI + Vehicle**  **(Saline)** | ***Drp1^C644S/+^***  **MI + GSSG**  **(30 mg/kg/day)** |
| --- | --- | --- | --- | --- |
| n | 5 | 5 | 5 | 6 |
| EDV  (µL) | 100.7 ± 13.7* | 105.8 ± 22.5** | 150.9 ± 18.0** | 133.5 ± 14.8** |
| EF (%)  4D-Mode | 18.9 ± 1.7** | 27.8 ± 3.5** | 14.4 ± 2.0** | 16.7 ± 1.5**## |
| HR  (rpm) | 487 ± 16 | 517 ± 22 | 513 ± 24 | 517 ± 18 |
| LVAWd  (mm) | 0.42 ± 0.02** | 0.55 ± 0.07* | 0.55 ± 0.03** | 0.57 ± 0.05** |
| LVPWd  (mm) | 0.82 ± 0.05 | 1.01 ± 0.08 | 0.9 ± 0.10 | 0.87 ± 0.11 |
|  | | | | |
| LVIDd  (mm) | 5.76 ± 0.30** | 5.63 ± 0.18** | 6.39 ± 0.26** | 6.15 ± 0.21** |
| LVIDs  (mm) | 5.14 ± 0.27** | 4.64 ± 0.21** | 5.81 ± 0.24** | 5.58 ± 0.23**# |
| EF (%)  M-Mode | 23.0 ± 1.7** | 36.5 ± 2.7**†† | 19.6 ± 1.6** | 20.1 ± 1.5**## |
| FS  (%) | 10.7 ± 0.8** | 17.8 ± 1.4**†† | 9.1 ± 0.8** | 9.3 ± 0.7**## |

EDV: end diastolic volume; EF: ejection fraction; HR: heart rate; LVAWd: LV anterior wall thickness at end diastole; LVPWd: LV posterior wall thickness at end diastole; LVIDd: LV internal diameter at end diastole; LVIDs: LV internal diameter at end systole; FS: fractional shortening. Data are shown as mean ± s.e.m. *P < 0.05, **P < 0.01 vs Sham within each group. †P < 0.05, ††P < 0.01 vs MI + Vehicle within each strain. #P < 0.05, ##P < 0.01 vs *Drp1^+/+^* MI + GSSG. Significance was determined using two-way ANOVA followed by Tukey’s multiple comparison test.

Supplementary Table 7. List of antibodies for western blot and immunostaining

|  | Antigen | Host | Supplier | Catalog No. | Dilution | Usage |
| --- | --- | --- | --- | --- | --- | --- |
| 1st | Glutathione (D8) | Mouse | abcam | ab19534 | 1:3,000  1:300 | WB  PLA |
|  | Drp1 | Mouse | BD Transduction | 611112 | 1:4,000 | WB |
|  | Drp1 (H-300) | Rabbit | Santa Cruz | sc-32898 | 1:100 | PLA |
|  | Filamin 1 (E-3) | Mouse | Santa Cruz | sc-17749 | 1:300 | WB |
|  | GAPDH | Mouse | Fujifilm | 014-25524 | 1:3,000 | WB |
|  | DYKDDDDK | Mouse | Fujifilm | 014-22383 | 1:3,000 | WB |
|  | c-Myc | Mouse | Fujifilm | 011-21874 | 1:2,000 | WB |
|  | p-53 | Rabbit | Cell Signaling | 9282 | 1:500  1:500 | IF  IHC |
|  | HIF-1alpha (D1S7W) | Rabbit | Cell Signaling | 36169 | 1:1,000 | WB |
|  | Sarcomeric alpha actinin | Mouse | abcam | ab9465 | 1:1,000  1:1,000 | IF  IHC |
|  | 4-HNE | Mouse | JaICA | MHN-100P | 1:50 | IHC |
| 2nd | Mouse IgG, HRP-linked | Goat | Cell Signaling | 7076 | 1:8,000 | WB |
|  | Rabbit IgG, HRP-linked | Goat | Cell Signaling | 7074 | 1:8,000 | WB |
|  | Mouse IgG, CF488A | Donkey | Biotium | 20014 | 1:1,000  1;1,000 | IF  IHC |
|  | Mouse IgG, CF594 | Donkey | Biotium | 20115 | 1:1,000  1;1,000 | IF  IHC |
|  | Rabbit IgG, CF488 | Donkey | Biotium | 20015 | 1:1,000  1;1,000 | IF  IHC |
|  | Rabbit IgG, CF596 | Donkey | Biotium | 20152 | 1:1,000  1;1,000 | IF  IHC |

IF, immunofluorescence; PLA, proximity ligation assay; WB, western blotting;

IHC, immunohistochemistry

Supplementary Table 8. The m/z of each modification in target peptides used for the proteomic analysis.

| Position | Peptide | Modification | m/z |
| --- | --- | --- | --- |
| 612-618 | DCEVIER | CysS-AM | 460.7115 |
|  |  | CysSS-AM | 476.6978 |
|  |  | CysS-SG | 584.735 |
|  |  | CysS-SSG | 600.7216 |
|  |  | CysS-SSSG | 616.7074 |
|  |  | CysS-AM-HPE | 520.7406 |
|  |  | CysSS-AM-HPE | 536.7264 |
|  |  | CysSSS-AM-HPE | 552.7122 |
|  |  | CysSSSS-AM-HPE | 568.6986 |
| 299-305 | DCLPELK | CysS-AM | 437.722 |
|  |  | CysS-SG | 561.7458 |
|  |  | CysS-SSG | 577.7308 |
|  |  | CysS-SSSG | 593.7168 |
|  |  | CysS-AM-HPE | 497.7496 |
|  |  | CysSS-AM-HPE | 513.7362 |
|  |  | CysSSS-AM-HPE | 529.7224 |
|  |  | CysSSSS-AM-HPE | 545.7087 |
| 340-353 | FATEYCNTIEGTAK | CysS-AM | 802.8679 |
|  |  | CysS-SG | 926.8919 |
|  |  | CysS-SSG | 942.8788 |
|  |  | CysS-SSSG | 958.8648 |
|  |  | CysS-AM-HPE | 862.897 |
|  |  | CysSS-AM-HPE | 878.8828 |
|  |  | CysSSS-AM-HPE | 894.8686 |
|  |  | CysSSSS-AM-HPE | 910.8546 |
| 354-365 | YIETSELCGGAR | CysS-AM | 678.3179 |
|  |  | CysS-SG | 802.3416 |
|  |  | CysS-SSG | 818.3277 |
|  |  | CysS-SSSG | 834.3136 |
|  |  | CysS-AM-HPE | 738.3465 |
|  |  | CysSS-AM-HPE | 754.3328 |
|  |  | CysSSS-AM-HPE | 770.3191 |
|  |  | CysSSSS-AM-HPE | 786.3054 |
| 366-376 | ICYIFHETFGR | CysS-AM | 481.5692 |
|  |  | CysS-SG | 564.2515 |
|  |  | CysS-SSG | 574.9086 |
|  |  | CysS-SSSG | 585.5658 |
|  |  | CysS-AM-HPE | 521.5878 |
|  |  | CysSS-AM-HPE | 532.2456 |
|  |  | CysSSS-AM-HPE | 542.9033 |
|  |  | CysSSSS-AM-HPE | 553.5611 |
| 431-441 | CVELVHEEMQR | CysS-AM | 715.3324 |
|  |  | CysS-SG | 839.3552 |
|  |  | CysS-SSG | 855.3435 |
|  |  | CysS-SSSG | 871.3293 |
|  |  | CysS-AM-HPE | 775.3625 |
|  |  | CysSS-AM-HPE | 791.3485 |
|  |  | CysSSS-AM-HPE | 807.3345 |
|  |  | CysSSSS-AM-HPE | 823.3205 |
| 442-456 | IIQHCSNYSTQELLR | CysS-AM | 621.6484 |
|  |  | CysS-SG | 703.9961 |
|  |  | CysS-SSG | 714.9879 |
|  |  | CysS-SSSG | 725.6456 |
|  |  | CysS-AM-HPE | 661.3334 |
|  |  | CysSS-AM-HPE | 672.3245 |
|  |  | CysSSS-AM-HPE | 682.982 |
|  |  | CysSSSS-AM-HPE | 693.6395 |
| 460-473 | LHDAIVEVVTCLLR | CysS-AM | 819.4573 |
|  |  | CysS-SG | 943.9826 |
|  |  | CysS-SSG | 959.9682 |
|  |  | CysS-SSSG | 975.9542 |
|  |  | CysS-AM-HPE | 879.9869 |
|  |  | CysSS-AM-HPE | 895.9731 |
|  |  | CysSSS-AM-HPE | 911.9593 |
|  |  | CysSSSS-AM-HPE | 927.9455 |
| 498-516 | HPDFADACGLMNNNIEEQR | CysS-AM | 744.3277 |
|  |  | CysS-SG | 827.0082 |
|  |  | CysS-SSG | 838.0001 |
|  |  | CysS-SSSG | 848.6574 |
|  |  | CysS-AM-HPE | 784.3426 |
|  |  | CysSS-AM-HPE | 795.3367 |
|  |  | CysSSS-AM-HPE | 805.9941 |
|  |  | CysSSSS-AM-HPE | 816.6515 |

Mass: AM, iodoacetamide adduct, 58.0293; AM-HPE, HPE-IAM adduct, 178.0868

**Supplementary methods.**

***Generation of Drp1^C644S^ knock-in mouse.*** The *Drp1^C644S^* knock-in mice were generated using the CRISPR/Cas9 method. We synthesized a ribonuclease protein (RNP) complex composed of Cas9 protein and a guide RNA (5′- CUCACAAUCUCGCUGUUCUCGUUUUAGAGCU AUGCU-3′), which causes a double-strand break. Pronuclear-stage zygotes were recovered from C57BL/6 female mice superovulated with eCG/hCG and mated with C57BL/6 male mice. The RNP complex and the above single-stranded oligodeoxynucleotide (CTTTTAGCCAGTT CCAGTTGCAAGAAAACTGTCTGCCCGAGAACAGCGAGATTCGGAGGTTATTGAAAGACTTATCAAATCATATTTTCT) were co-injected into a pronucleus of the zygotes. The surviving zygotes were transferred into the oviductal ampullae of pseudopregnant ICR recipient mouse (≥ 10 weeks old) anesthetized with isoflurane 2–2.5% in oxygen at 0.5 days post-coitum (dpc) (< 25 embryos per recipient). To select the desired founder mice, obtained F0 pups were screened by the genomic PCR analysis (Primer set, Fwd: 5′-CTACAAGTGAACCAAGTTAG TAGCAGAC-3′, Rev: 5′-GTCAACAGACTAAGTCTTGCTATCACAG-3′) and subsequent direct sequencing analyses. Then, the founder mice harboring the knock-in allele were backcrossed with C57BL/6 mice.

***Purification of Drp1 protein for mass spectrometry.*** N-terminal His-tagged Drp1 (His-Drp1) and C-terminal FLAG-tagged Drp1 (Drp1-FLAG) were purified from *Escherichia coli* or HeLa cells, respectively. For the analysis of supersulfide-related modification with recombinant Drp1 from *E. coli*, the cell pellet was lysed with Lysis buffer A (40 mM phosphate pH7, 150 mM NaCl, 1% Triton X-100 and protease inhibitor cocktail) with 10 mM Imidazole (for His-Drp1). The lysate was centrifuged and supernatant was recovered. His-Drp1 was precipitated using Ni-NTA agarose and eluted by Elution buffer (40 mM phosphate pH7.4 and 150 mM NaCl) with 300 mM Imidazole. For the analysis of supersulfide and other cysteine modifications with Drp1 occurring in the cellular context, HeLa cells transfected with Drp1-FLAG were starved for 1 day and harvested. Cell pellet was lysed with Lysis buffer A with 3 mM tyrosine and 2 mM HPE-IAM. Tyrosine was added to protect polysulfide bridges of protein supersulfides from hydrolysis. To avoid artificial modification of polysulfidated cysteine, thiol groups are labeled with HPE-IAM during the purification step. Drp1-FLAG was precipitated using M2-agarose and eluted by 300 µg/ml FLAG peptide, following additional elution with 100 mM glycine pH3 (neutralize sample with 100 mM Tris pH7.5 after elusion). FLAG peptide elution and glycine elution samples were mixed and used for mass spectrometry.

***Proteomic analysis of cysteine residues in Drp1.*** The supersulfide proteome analysis of Drp1 was performed according to our previous reports with slight modifications^1, 2^. Purified Drp1 (7 µM; from *E. coli*) was incubated with 3 mM GSSG at 37 ºC for 1 h. Following desalting via the PD SpinTrap G-25 column, the sample was alkylated with 10 mM iodoacetamide (IAM) in 0.02% ProteaseMAX surfactant at 37ºC for 10 min. The alkylated sample was digested with 27 µg/ml Trypsin Gold at 37ºC for 3 h. Drp1 (2.5 µM) from HeLa cells pretreated with HPE-IAM was further alkylated with 10 mM HPE-IAM in 0.02% ProteaseMAX surfactant at 37ºC for 20 min. The alkylated sample was digested with 27 µg/ml Trypsin Gold at 37ºC for 3 h. To examine the effect of CysSH polysulfidation on S-glutathionylation efficiency by GSSG, purified His-Drp1 (6.3 µM; from *E. coli*) was incubated with 300 µM Na_2_S_4_ in anaerobic conditions at 37 ºC for 30 min. Immediately after desalting via the PD SpinTrap G-25 column, the sample was reacted with 1 mM GSSG at 37 ºC for 1 h, followed by alkylation with 10 mM HPE-IAM in 0.02% ProteaseMAX surfactant at 37ºC for 10 min. The alkylated protein was then digested with 27 µg/ml Trypsin Gold at 37ºC for 3 h. Samples were analyzed using liquid chromatography-electrospray ionization-quadrupole time-of-flight tandem mass spectrometry (LC-ESI-Q-TOF MS/MS). LC-ESI-Q-TOF analysis was performed using 6545XT AdvanceBio LC/Q-TOF (Agilent Technologies) connected to the Agilent HPLC system. The modification analysis of the cysteine residue was performed using Agilent MassHunter BioConfirm software. The modification levels of target peptides were detected by monitoring the m/z of each modification. The m/z of each modification in target peptides were indicated at Supplementary Table 8. The digestion efficiency of the protein by trypsin was normalized with TLESVDPLGGLNTIDILTAIR peptide (m/z 1106.6199). The modification levels of the peptide containing the cysteine were assessed by determining the relative ratio of the intensity of each modification against that of CysS-AM or CysS-AM-HPE containing peptide (AM, iodoacetamide adduct; AM-HPE, HPE-IAM adduct). The CysS-AM or CysS-AM-HPE containing peptide (for S-glutathionylation or polysulfidation, respectively), samples subjected to trypsin digestion followed by reduction with TCEP and subsequent alkylation with IAM or HPE-IAM, was used to measure peptides corresponding to each modification.

***MD simulation and computational analysis.*** The dynamin-related protein 1 (Drp1) was obtained from the protein data bank (PDB ID: 5WP9). In this study, the GTPase domain of Drp1 was excluded. Missing residue at the variable loop (residue 503-637) predicted as an intrinsically disordered region (IDR) was built using the deep-learning algorithm, AlphaFold 2. However, the IDR expected by AlphaFold 2 was a linear structure. The IDRs of Drp1 were subsequently generated using all-atom molecular dynamics simulations implemented in AMBER 20 package software. The initial structure of Drp1 at the middle and variable region was protonated at the neutral condition (pH=7) using PDB2PQT. Four Na^2+^ ions, hydrogen atoms, and TIP3P water molecules were added to the periodic boundary condition (PBC) box with a 12 Å cutoff. The minimization was performed using SANDER and PMEMD.CUDA with ff19SB AMBER force field for the standard amino acid residues according to the standard protocol. The system was minimized with 5,000 steps for steepest descent (2,500 steps) and conjugate gradient (2,500 steps) minimizations. Then, the system was heated from 0 to 300 K for 100 ps with an NVT ensemble. Next, the NPT equilibration was continued and simulated at the same temperature for 200 ns. A representative model was extracted from the whole MD trajectory using average structure analysis. The geometry and stereochemistry of the representative model were verified using the Ramachandran plot. This model was considered a CysSH system.

Models with a cysteine persulfide (CysSSH) at C644, the C644W mutation (CW), C644 bound to glutathione (GSH), and E640A mutation (EA) were constructed based on the CysSH structure derived from the previous step. First constructed dipeptides as Ace-CysSSH-NMe and Ace-Cys-GSH-NMe using GaussView 6. Protein backbone parameters were employed from the general Amber force field 2 (GAFF2). For the constructed cysteine derivatives, the structural optimization was performed at the B3LYP/6-31G* level. Consequently, the electrostatic potential (ESP) charges were calculated based on the optimized structure at the B3LYP/6-31G* level. The restrained electrostatic potential (RESP) charges were generated according to the ESP-fit charges model. Sidechain parameters of CysSSH and GSH were produced using an antechamber module in AmberTools20. For per-mutation, residue Cys644 and Glu640 were replaced by Trp and Ala using Dunbrack 2010 rotamer library implemented in Chimera USCF. The topology and coordinate files of all systems were created for MD simulations according to the representative model using the tLeap program of AmberTools20. The energy minimization and the MD simulations were performed in accordance with the previous step.

The MD trajectory analyses were performed using cpptraj module implemented in AMBER20 package program. The structural stability of Drp1 was analyzed from the MD trajectories of 5 systems, CysSH: depolysulfidated Cys644, CysSSH: polysulfidated Cys644, CW: C644W, GSH: S-glutathionylated Cys644, and EA: E640A + depolysulfidated Cys644. One of the criteria used to evaluate the system stability is a radius of gyration (*R_g_*) that can reveal the steadiness of the Drp1 at the stalk domain and the influence on the phosphorylation of S637. Root mean square fluctuation (RMSF) was used to identify conformational changes in these proteins. Hydrogen bonding interaction was computed to introduce the critical interacted residue for each system. Principal component analysis (PCA), the empirical C-matrix, can be used to estimate protein fluctuation and mobility along the trajectory. In this study, the range of 400-500 ns-MD trajectories of these 3 systems; CysSH, CysSSH, and EA, was used to analyze and compare the dynamics of VD region.

***Preparation of cigarette sidestream smoke (CSS).*** CSS was prepared as followed^3^. CSS was generated by spontaneous combustion of five cigarettes (Seven Star: tar; 14 mg, nicotine; 1.2 mg, Japan Tobacco Co.), and then trapped into 100 ml DMEM by bubbling using a dry vacuum pump. The generated CSS was designated 100% CSS.

1. Matsunaga T*, et al.* Supersulphides provide airway protection in viral and chronic lung diseases. *Nat Commun* **14**, 4476 (2023).

2. Kasamatsu S*, et al.* Supersulfide catalysis for nitric oxide and aldehyde metabolism. *Sci Adv* **9**, eadg8631 (2023).

3. Ibuki Y, Toyooka T, Zhao X, Yoshida I. Cigarette sidestream smoke induces histone H3 phosphorylation via JNK and PI3K/Akt pathways, leading to the expression of proto-oncogenes. *Carcinogenesis* **35**, 1228-1237 (2014).
