# Supplementary material for: Polysulfur-based bulking of dynamin-related protein 1 prevents ischemic sulfide catabolism and heart failure in mice
Source: Nat Commun. 2025 Jan 2;16:276. doi: 10.1038/s41467-024-55661-5 (PMC11695708; doi:10.1038/s41467-024-55661-5)
Supplement: Supplementary file 2 — Reporting Summary [file 41467_2024_55661_MOESM2_ESM.pdf]

nature portfolio

Corresponding author(s): Motohiro Nishida

Last updated by author(s): Nov 16, 2024

Reporting Summary

Nature Portfolio wishes to improve the reproducibility of the work that we publish. This form provides structure for consistency and transparency in reporting. For further information on Nature Portfolio policies, see our Editorial Policies and the Editorial Policy Checklist.

Statistics

For all statistical analyses, confirm that the following items are present in the figure legend, table legend, main text, or Methods section.

n/a	Confirmed	
☐	☒	The exact sample size (*n*) for each experimental group/condition, given as a discrete number and unit of measurement
☐	☒	A statement on whether measurements were taken from distinct samples or whether the same sample was measured repeatedly
☐	☒	The statistical test(s) used AND whether they are one- or two-sided<br>*Only common tests should be described solely by name; describe more complex techniques in the Methods section.*
☐	☒	A description of all covariates tested
☐	☒	A description of any assumptions or corrections, such as tests of normality and adjustment for multiple comparisons
☐	☒	A full description of the statistical parameters including central tendency (e.g. means) or other basic estimates (e.g. regression coefficient) AND variation (e.g. standard deviation) or associated estimates of uncertainty (e.g. confidence intervals)
☐	☒	For null hypothesis testing, the test statistic (e.g. *F*, *t*, *r*) with confidence intervals, effect sizes, degrees of freedom and *P* value noted<br>*Give P values as exact values whenever suitable.*
☒	☐	For Bayesian analysis, information on the choice of priors and Markov chain Monte Carlo settings
☒	☐	For hierarchical and complex designs, identification of the appropriate level for tests and full reporting of outcomes
☒	☐	Estimates of effect sizes (e.g. Cohen's *d*, Pearson's *r*), indicating how they were calculated

Our web collection on statistics for biologists contains articles on many of the points above.

Software and code

Policy information about availability of computer code

Data collection	No software was used for data collection.
Data analysis	GraphPad 9.0 (GraphPad Software, Lajolla, CA) for statistical analysis. ImageJ (National Institutes of Health) for imaging data analysis. ImageQuant LT for quantification of blot bands. G*Power3.1.9.2 software for sample size calculation. MassHunter BioConfirm (Agilent, CA) for Mass spectrometry-based analysis of cysteine modification. AlphaFold2 for molecular modeling. For the molecular modeling, the parameters for special amino acids and glutathione were generated using Gaussian 16. Molecular dynamics simulations were performed using the AMBER 20 package program. The 3D structure was visualized using VMD (version 1.9.3) and UCSF Chimera 1.17.1.

For manuscripts utilizing custom algorithms or software that are central to the research but not yet described in published literature, software must be made available to editors and reviewers. We strongly encourage code deposition in a community repository (e.g. GitHub). See the Nature Portfolio guidelines for submitting code & software for further information.

Data

Policy information about availability of data

All manuscripts must include a data availability statement. This statement should provide the following information, where applicable:

- Accession codes, unique identifiers, or web links for publicly available datasets
- A description of any restrictions on data availability
- For clinical datasets or third party data, please ensure that the statement adheres to our policy

All data generated and analyzed in this study are included in this article and its Supplementary information files. The mass spectrometry proteomics data generated

in this study have been deposited in the ProteomeXchange Consortium via the PRIDE partner repository under accession code PXD053562. Source data are provided with this paper.

Research involving human participants, their data, or biological material

Policy information about studies with human participants or human data. See also policy information about sex, gender (identity/presentation), and sexual orientation and race, ethnicity and racism.

Reporting on sex and gender	*Use the terms sex (biological attribute) and gender (shaped by social and cultural circumstances) carefully in order to avoid confusing both terms. Indicate if findings apply to only one sex or gender; describe whether sex and gender were considered in study design; whether sex and/or gender was determined based on self-reporting or assigned and methods used. Provide in the source data disaggregated sex and gender data, where this information has been collected, and if consent has been obtained for sharing of individual-level data; provide overall numbers in this Reporting Summary. Please state if this information has not been collected. Report sex- and gender-based analyses where performed, justify reasons for lack of sex- and gender-based analysis.*
Reporting on race, ethnicity, or other socially relevant groupings	*Please specify the socially constructed or socially relevant categorization variable(s) used in your manuscript and explain why they were used. Please note that such variables should not be used as proxies for other socially constructed/relevant variables (for example, race or ethnicity should not be used as a proxy for socioeconomic status). Provide clear definitions of the relevant terms used, how they were provided (by the participants/respondents, the researchers, or third parties), and the method(s) used to classify people into the different categories (e.g. self-report, census or administrative data, social media data, etc.) Please provide details about how you controlled for confounding variables in your analyses.*
Population characteristics	*Describe the covariate-relevant population characteristics of the human research participants (e.g. age, genotypic information, past and current diagnosis and treatment categories). If you filled out the behavioural & social sciences study design questions and have nothing to add here, write "See above."*
Recruitment	*Describe how participants were recruited. Outline any potential self-selection bias or other biases that may be present and how these are likely to impact results.*
Ethics oversight	*Identify the organization(s) that approved the study protocol.*

Note that full information on the approval of the study protocol must also be provided in the manuscript.

Field-specific reporting

Please select the one below that is the best fit for your research. If you are not sure, read the appropriate sections before making your selection.

- [x] Life sciences
- [ ] Behavioural & social sciences
- [ ] Ecological, evolutionary & environmental sciences

For a reference copy of the document with all sections, see nature.com/documents/nr-reporting-summary-flat.pdf

Life sciences study design

All studies must disclose on these points even when the disclosure is negative.

Sample size	G*Power3.1.9.2 software was used to calculate the sample size for each group.
Data exclusions	No data were excluded from the manuscript.
Replication	As reported in the figure legends, the findings were reliably reproduced.
Randomization	Laboratory animals were randomly assigned to experimental groups. The order of treatment administration was also randomized. All cell samples were randomly selected for experiments.
Blinding	All animal samples were studied, and analysis was carried out in a blinded manner. Other Investigations were not blinded during data acquisition. Measurements and data reported did not require subjective judgment or interpretation from the investigators.

Reporting for specific materials, systems and methods

We require information from authors about some types of materials, experimental systems and methods used in many studies. Here, indicate whether each material, system or method listed is relevant to your study. If you are not sure if a list item applies to your research, read the appropriate section before selecting a response.

Materials & experimental systems

n/a	Involved in the study
☐	☒ Antibodies
☐	☒ Eukaryotic cell lines
☒	☐ Palaeontology and archaeology
☐	☒ Animals and other organisms
☒	☐ Clinical data
☒	☐ Dual use research of concern
☒	☐ Plants

Methods

n/a	Involved in the study
☒	☐ ChIP-seq
☒	☐ Flow cytometry
☒	☐ MRI-based neuroimaging

Antibodies

Antibodies used

mouse monoclonal anti-glutathione (D8) antibody (1:3,000 dilution for WB, 1:300 for PLA, ab19534, abcam), mouse monoclonal anti-DLP1 antibody (1:4,000 for WB, 611112, BD Transduction), mouse monoclonal anti-GAPDH (1:3,000 for WB, 014-25524, Fujifilm), mouse monoclonal anti-DYKDDDDK antibody (1:3,000 for WB, 014-22383, Fujifilm), mouse monoclonal anti-c-Myc antibody (1:2,000 for WB, 011-21874, Fujifilm), mouse monoclonal anti-Filamin 1 (E-3) antibody (1:300 for WB, sc-17749, Santa Cruz), rabbit polyclonal anti-Drp1 (H-300) antibody (1:100 for PLA, sc-32898, Santa Cruz), rabbit polyclonal anti-p53 antibody (1:500 for IF and IHC, #9282, Cell Signaling), mouse monoclonal anti-Sarcomeric alpha actinin antibody (1:1,000 for IF and IHC, ab9465, abcam), rabbit monoclonal anti-HIF-1alpha (D1S7W) antibody (1:1,000 for WB, #36169, Cell Signaling), mouse monoclonal anti-4HNE antibody (1:50 for IHC, MHN-100P, JaICA).

Validation

All antibodies were well-recognized clones in the field and validated by the manufacturers. These antibodies are further validated and routinely used in our lab.
Abbreviation for species reactivity: H-human, M-mouse, R-rat, Mk-monkey, B-Bovine, Pg-Pig
Abbreviation for application: WB- western blotting, IP-Immunoprecipitation, IF- Immunofluorescence, IHC- Immunohistochemistry,

Glutathione (ab19534, Abcam)
Application: IF
Web: https://www.abcam.co.jp/products/primary-antibodies/glutathione-antibody-d8-ab19534.html

Drp1 (611112, BD Transduction)
Species reactivity: H, M
Application: IF, WB
Web: https://www.bdbiosciences.com/en-us/products/reagents/microscopy-imaging-reagents/immunofluorescence-reagents/purified-mouse-anti-dlp1.611112

GAPDH (014-25524, Fujifilm)
Species reactivity: H, M, R, Pg
Application: IP, WB
Web: https://labchem-wako.fujifilm.com/jp/product/detail/W01W0101-2552.html

DYKDDDDK (014-22383, Fujifilm)
Application: IP, WB
Web: https://labchem-wako.fujifilm.com/jp/product/detail/W01W0101-2238.html

c-Myc (011-21874, Fujifilm)
Application: IP, WB
Web: https://labchem-wako.fujifilm.com/jp/product/detail/W01W0101-2187.html

Filamin 1 (sc-17749, Santa cruz)
Species reactivity: H, M, R
Application: IF, WB, IP, IHC
Web: https://www.scbt.com/ja/p/filamin-1-antibody-e-3?srsltid=AfmBOorKIlrLB42tLLbflo4rbUKPTc_zeQsnKo0Z8xPrTKu9eUlyf5wE

Drp1 (sc-32898, Santa cruz)
Species reactivity: H, M, R
Application: IF, WB, IP, IHC
Web: https://www.scbt.com/ja/p/drp1-antibody-h-300

p53 (#9282, Cell Signaling)
Species reactivity: H, Mk
Application: WB, IP
Web: https://www.cellsignal.jp/products/primary-antibodies/p53-antibody/9282

Sarcomeric Alpha Actinin (ab9465, Abcam)

Species reactivity: H, R
Application: WB, IHC
Web: https://www.abcam.co.jp/products/primary-antibodies/sarcomeric-alpha-actinin-antibody-ea-53-ab9465.html

HIF-1alpha (#36169, Cell Signaling)
Species reactivity: H, M, Mk
Application: WB, IP, IF
Web: https://www.cellsignal.jp/products/primary-antibodies/hif-1a-d1s7w-xp-rabbit-mab/36169

4-HNE (MHN-100P, JaICA)
Application: WB, IHC
Web: https://www.jaica.com/e/products_lipid_4hne_ab.html

Eukaryotic cell lines

Policy information about cell lines and Sex and Gender in Research

Cell line source(s)	293T cells (ATCC Cat#CRL-3216), HeLa cells (ATCC Cat#CCL-2).
Authentication	Cell lines were not authenticated.
Mycoplasma contamination	All cell lines were tested negatively for mycoplasma contamination.
Commonly misidentified lines (See ICLAC register)	No commonly misidentified line were used in this study.

Animals and other research organisms

Policy information about studies involving animals; ARRIVE guidelines recommended for reporting animal research, and Sex and Gender in Research

Laboratory animals	Drp1 C644S knockin mice were made of C57BL/6J. Seven-week-old male C57BL/6J mice and Sprague-Dawley rat pups (male and female) on postnatal day 1-2 were purchased from Japan SLC, Inc. All mice were kept in plastic cages in a climate-controlled animal room with a 12-hour light/dark cycle, and then male mice aged 8-10 weeks were used for experiments.
Wild animals	This study didn't involve wild animals.
Reporting on sex	Male mice were used in this study.
Field-collected samples	This study didn't involve sample collected from field.
Ethics oversight	All protocols using mice and rats were reviewed and approved by the ethics committees at the National Institutes of Natural Sciences and Kyushu University and carried out in accordance with their guidelines.

Note that full information on the approval of the study protocol must also be provided in the manuscript.

Plants

Seed stocks	*Report on the source of all seed stocks or other plant material used. If applicable, state the seed stock centre and catalogue number. If plant specimens were collected from the field, describe the collection location, date and sampling procedures.*
Novel plant genotypes	*Describe the methods by which all novel plant genotypes were produced. This includes those generated by transgenic approaches, gene editing, chemical/radiation-based mutagenesis and hybridization. For transgenic lines, describe the transformation method, the number of independent lines analyzed and the generation upon which experiments were performed. For gene-edited lines, describe the editor used, the endogenous sequence targeted for editing, the targeting guide RNA sequence (if applicable) and how the editor was applied.*
Authentication	*Describe any authentication procedures for each seed stock used or novel genotype generated. Describe any experiments used to assess the effect of a mutation and, where applicable, how potential secondary effects (e.g. second site T-DNA insertions, mosiacism, off-target gene editing) were examined.*
